# A multiphase program for malaria elimination in southern Mozambique (the Magude project): A before-after study

**DOI:** 10.1371/journal.pmed.1003227

**Published:** 2020-08-14

**Authors:** Beatriz Galatas, Francisco Saúte, Helena Martí-Soler, Caterina Guinovart, Lidia Nhamussua, Wilson Simone, Humberto Munguambe, Camilo Hamido, Júlia Montañà, Olinda Muguande, Francois Maartens, Fabião Luis, Krijn Paaijmans, Alfredo Mayor, Quique Bassat, Clara Menéndez, Eusebio Macete, Regina Rabinovich, Pedro L. Alonso, Baltazar Candrinho, Pedro Aide

**Affiliations:** 1 ISGlobal, Hospital Clínic—Universitat de Barcelona, Barcelona, Spain; 2 Centro de Investigação em Saúde de Manhiça, Maputo, Mozambique; 3 Fundação para o Desenvolvimento da Comunidade, Maputo, Mozambique; 4 Good Bye Malaria, Johannesburg, South Africa; 5 School of Life Sciences, Center for Evolution and Medicine, Biodesign Center for Immunotherapy, Vaccines and Virotherapy, Arizona State University, Tempe, United States of America; 6 CIBER Epidemiología y Salud Pública (CIBERESP), Madrid, Spain; 7 ICREA, Pg. Lluís Companys 23, Barcelona, Spain; 8 National Institute of Health, Ministry of Health, Maputo, Mozambique; 9 Harvard T.H. Chan School of Public Health, Boston, Massachusetts, United States of America; 10 National Malaria Control Program, Ministry of Health, Maputo, Mozambique; Mahidol Oxford Tropical Medicine Research Unit, Faculty of Tropical Medicine, Mahidol University, THAILAND

## Abstract

**Background:**

Malaria eradication remains the long-term vision of the World Health Organization (WHO). However, whether malaria elimination is feasible in areas of stable transmission in sub-Saharan Africa with currently available tools remains a subject of debate. This study aimed to evaluate a multiphased malaria elimination project to interrupt *Plasmodium falciparum* malaria transmission in a rural district of southern Mozambique.

**Methods and findings:**

A before-after study was conducted between 2015 and 2018 in the district of Magude, with 48,448 residents living in 10,965 households. Building on an enhanced surveillance system, two rounds of mass drug administrations (MDAs) per year over two years (phase I, August 2015–2017), followed by one year of reactive focal mass drug administrations (rfMDAs) (phase II, September 2017–June 2018) were deployed with annual indoor residual spraying (IRS), programmatically distributed long-lasting insecticidal nets (LLINs), and standard case management. The four MDA rounds covered 58%–72% of the population, and annual IRS reported coverage was >70%. Yearly parasite surveys and routine surveillance data were used to monitor the primary outcomes of the study—malaria prevalence and incidence—at baseline and annually since the onset of the project. Parasite prevalence by rapid diagnostic test (RDT) declined from 9.1% (95% confidence interval [CI] 7.0–11.8) in May 2015 to 2.6% (95% CI 2.0–3.4), representing a 71.3% (95% CI 71.1–71.4, *p* < 0.001) reduction after phase I, and to 1.4% (95% CI 0.9–2.2) after phase II. This represented an 84.7% (95% CI 81.4–87.4, *p* < 0.001) overall reduction in all-age prevalence. Case incidence fell from 195 to 75 cases per 1,000 during phase I (61.5% reduction) and to 67 per 1,000 during phase II (65.6% overall reduction). Interrupted time series (ITS) analysis was used to estimate the level and trend change in malaria cases associated with the set of project interventions and the number of cases averted. Phase I interventions were associated with a significant immediate reduction in cases of 69.1% (95% CI 57.5–77.6, *p* < 0.001). Phase II interventions were not associated with a level or trend change. An estimated 76.7% of expected cases were averted throughout the project (38,369 cases averted of 50,005 expected). One malaria-associated inpatient death was observed during the study period. There were 277 mild adverse events (AEs) recorded through the passive pharmacovigilance system during the four MDA rounds. One serious adverse event (SAE) that resulted in death was potentially related to the drug. The study was limited by the incomplete coverage of interventions, the quality of the routine and cross-sectional data collected, and the restricted accuracy of ITS analysis with a short pre-intervention period.

**Conclusion:**

In this study, we observed that the interventions deployed during the Magude project fell short of interrupting *P*. *falciparum* transmission with the coverages achieved. While new tools and strategies may be required to eventually achieve malaria elimination in stable transmission areas of sub-Saharan Africa, this project showed that innovative mixes of interventions can achieve large reductions in disease burden, a necessary step in the pathway towards elimination.

**Trial registration:**

ClinicalTrials.gov NCT02914145.

## Abstract 2

Beatriz Galatas and colleagues evaluate a multi-intervention program for malaria prevention in southern Mozambique.

## Author summary

**Why was the study done?:**

- The World Health Organization (WHO) has the vision of malaria eradication.
- However, whether malaria elimination is possible in areas of stable transmission in sub-Saharan Africa remains a subject of debate.
- The Magude project was designed to evaluate the feasibility of malaria elimination using the prevention and treatment tools currently available, in a malaria endemic district of southern Mozambique.

**What did the researchers do and find?:**

- The project was divided into two phases aimed to drastically decrease the number of malaria cases to zero (phase I) and to sustain the gains for one year after phase I (phase II).
- The impact of the project was monitored through changes in the prevalence of infections in the community, the incidence of clinical cases detected at the health facilities (HFs), and the number of cases averted.
- Reductions of 84.7% of prevalence and 65.6% of incidence were observed during the project, which translated to an estimated 38,369 (76.7%) cases averted.

**What do these findings mean?:**

- Results from this project indicate that the implementation of a package of interventions in sub-Saharan Africa cannot interrupt malaria transmission, and therefore malaria elimination in these areas remains a long-term goal for which new tools and strategies may be required.
- In spite of this, the Magude project revealed that an intensive implementation of currently available tools recommended by WHO can achieve major reductions in malaria transmission and burden of disease.

## Introduction

Mozambique is one of the 10 countries with the highest malaria burden in the world, with parasite prevalence ranging from 3% in the south to >50% in the north of the country [1]. While the last decade witnessed significant reductions in the burden of malaria throughout the country, the gains have since stalled, and an increase in disease incidence [2,3] has been observed since 2014 [4]. Mozambique’s National Malaria Control Program (NMCP) has focused on increasing the coverage of long-lasting insecticidal nets (LLINs), improved case management, and surveillance systems throughout the country while aiming for elimination in the low endemic areas of the south through yearly rounds of universal indoor residual spraying (IRS) [5]. In fact, the south of Mozambique has historically experienced unsuccessful malaria control and elimination attempts using mainly IRS in the 1960s during the Global Malaria Eradication Program, and in the 2000s in the context of the Lubombo Spatial Development Initiative (LSDI) that aimed to eliminate malaria in South Africa and Eswatini [6].

The World Health Organization (WHO) Global Technical Strategy (GTS) for Malaria 2016–2030 calls for the generation of evidence to identify new tools and strategies to accelerate towards malaria elimination, as well as to refine approaches to better implement currently available tools [7]. The GTS further recommends that surveillance should be used as an intervention through the implementation of response strategies to malaria cases when transmission is low [7,8]. In this context, the Magude project was designed to evaluate the feasibility of malaria elimination in sub-Saharan Africa using a comprehensive mix of interventions recommended by the GTS [9], including a strengthened surveillance system, case management, vector control with LLINs and IRS, and mass drug administration (MDA). This article presents the impact evaluation of the project on *P*. *falciparum* malaria cases, and the changes observed in prevalence, incidence, and inpatient admissions and mortality in Magude district, southern Mozambique.

## Methods

### Study area

Magude district is a rural district located in the northwest of Maputo province, southern Mozambique (26° 02’ 00” south, 32° 17’ 00’ east), away from Mozambique’s main road. A baseline census conducted at the beginning of 2015 identified 48,448 residents living in 10,965 households. Malaria transmission is perennial with marked seasonality between November and April, coinciding with the rainy season. *P*. *falciparum* accounts for the majority of infections. Studies in nearby areas have identified *Anopheles funestus s*.*s*. as the most abundant vector responsible for the majority of transmission, followed by *Anopheles arabiensis* [10,11]. High levels of pyrethroid resistance have also been described in *A*. *funestus*, but not in *A*. *arabiensis* [12,13]. Artemether-lumefantrine, the national first-line antimalarial treatment, remains fully efficacious [14].The district has 27 community health workers (CHWs), nine rural health facilities (HFs), one referral health center with an inpatient ward in Magude Sede (main town), although the more severe cases are sometimes referred to the neighboring hospitals of Xinavane or Manhiça (Manhiça District). Magude’s baseline sociodemographic characteristics and health systems have been described elsewhere [15].

### Study design

**Fig 1:**
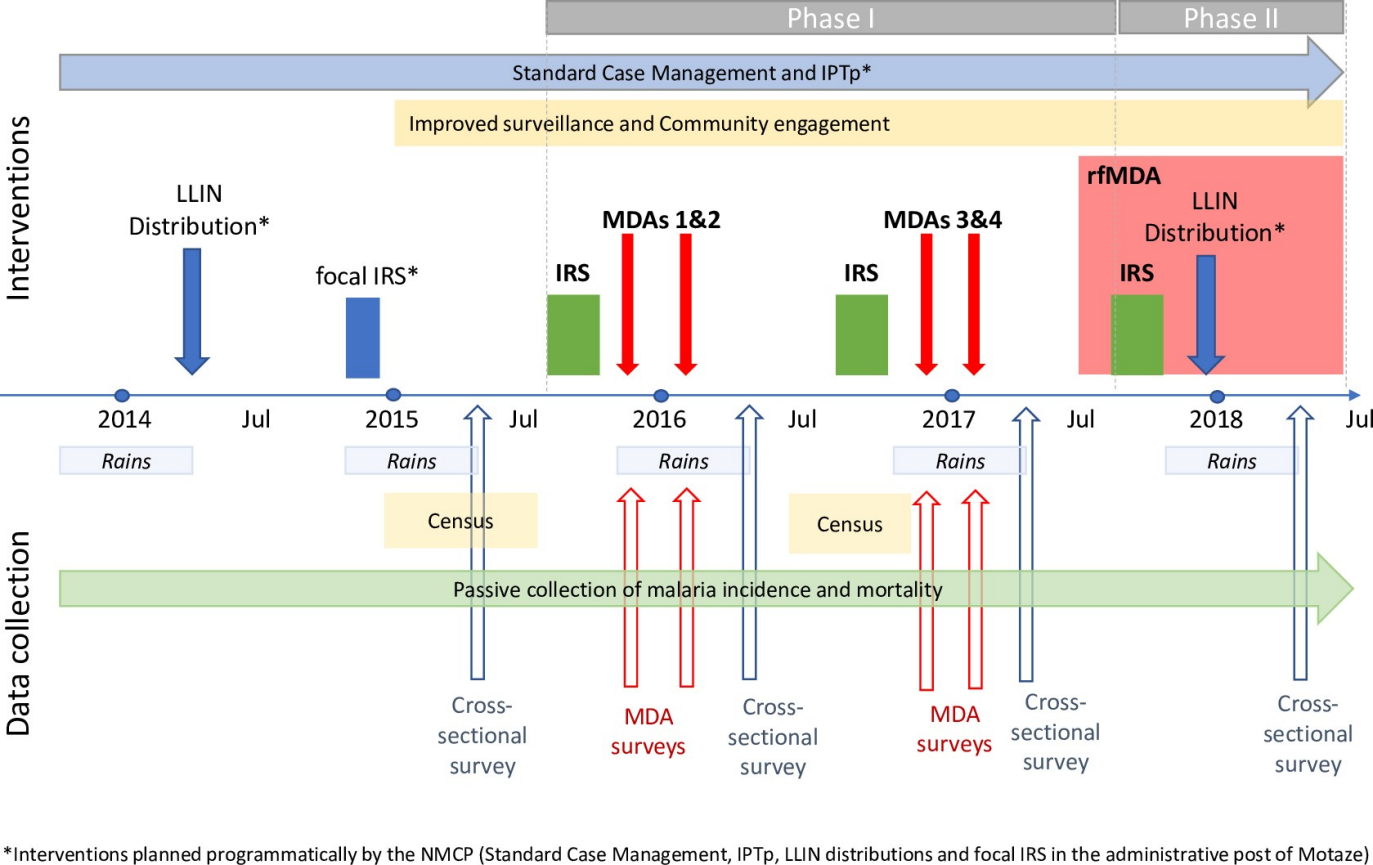
Project interventions and outcome evaluation timeline. Timing of the Magude project interventions and of the different data collection approaches used to measure and compare the primary outcomes of the project, and to estimate impact. IPTp, intermittent preventive treatment for pregnant women; IRS, indoor residual spraying; LLIN, long-lasting insecticidal net; MDA, mass drug administration; NMCP, National Malaria Control Program; rfMDA, reactive focal mass drug administration.

A before-after design was used to assess whether the combination of interventions would interrupt local transmission defined as zero indigenous clinical malaria cases. This study is reported as per the Strengthening the Reporting of Observational Studies in Epidemiology (STROBE) guidelines (S1 Checklist). The project was conducted jointly with the Ministry of Health (MoH) and district health authorities, building on the programmatic activities planned for the district [9]. Fig 1 shows the implementation timeline of interventions and the data collection methods.

### Interventions and study procedures

#### Programmatic interventions: Malaria case management, intermittent preventive treatment for pregnant women, and LLIN distributions

Malaria case management in Magude consisted of the standard of care provided by the national health system, which relied on passive detection and testing with a histidine-rich protein 2 (HRP2)-based rapid diagnostic test (RDT) of all patients presenting with a fever (axillary temperature ≥37.5°C or reported fever in the previous 24 hours) at the HFs or to CHWs (or “*Agente Polivalente Elemental*,” APE in Portuguese), and on treating the confirmed positives with artemether-lumefantrine. Monthly intermittent preventative treatment of pregnant women (IPTp) with sulfadoxine-pyrimethamine is provided through antenatal clinics (ANCs) at any of the HFs since the 13th gestational week. Magude also received 35,432 and 44,400 LLINs during the national distribution campaigns conducted by the NMCP in May of 2014 and December of 2017, respectively [16], and focal IRS in the subdistrict administrative area of Motaze in September of 2014.

#### Enhanced surveillance system

The reporting of district-level malaria data through the national monthly routine surveillance system using District Health Information System 2 (DHIS2) was expanded to all HFs and CHWs and strengthened through monthly meetings and data quality audits. Additionally, a DHIS2-based rapid reporting malaria surveillance system (RRS) was established in the district in January 2015 to provide weekly data for the project. The data submitted weekly included total number of outpatient visits, RDTs performed or slides read using microscopy, positive slides or RDTs, and cases treated, stratified by <5 and ≥5 years old (including malaria cases among pregnant women tested in the outpatient ward). Baseline malaria incidence weekly data were also retrospectively collected for 2013 and 2014 from the HF and CHWs registers. The monthly number of all-cause admissions, and admissions and deaths that were classified as malaria in the inpatient ward registers of the Magude Sede referral health center since 2011 were retrospectively collected.

An entomological surveillance platform was established at six sentinel sites to guide vector control strategies and assess their effectiveness. Vector species composition, densities, and insecticide resistance were monitored [9]. The entomological outcomes of this project will be presented in subsequent publications.

#### Community engagement

A community engagement campaign was rolled out in May of 2015 and continued until June of 2018 in collaboration with the District health authorities and Fundação para o Desenvolvimento da Comunidade (FDC). Information about vector control and MDA activities was shared in discussion groups with community leaders and the general population, as well as through the radio, public announcements, and in specific promotional events in schools, places of worship, or markets.

#### Indoor residual spraying

IRS was implemented by Goodbye Malaria (GBM) to all households in the district between August and October of 2015, and between September and November of 2016, 2017, and 2018. The insecticides used in 2015 were dichlorodiphenyltrichloroethane (DDT) for houses with thatched or mud walls (47%), and pirimiphos-methyl (Actellic 300 CS, Syngenta Crop Protection AG, Basel, Switzerland) for houses with concrete walls (53%). In 2016, 2017, and 2018, IRS was only conducted using Actellic.

#### Mass drug administration

Two rounds of MDA with dihydroartemisinin-piperaquine (DHAp, Alfasigma, Italy) separated by a period of 4–6 weeks were deployed starting at the beginning of the rainy season of 2015–2016 (November 2015 [MDA1] and January–February 2016 [MDA2]) and repeated one year later (December 2016 [MDA3] and January–February 2017 [MDA4]). MDAs were targeted to the de facto population of Magude, which included visitors and passers-by at the time of the MDA campaigns. MDAs were performed door to door in all households of Magude, as well as in markets and at populated venues, such as at the sugar cane plantation in the nearby area of Xinavane, where many residents of Magude work.

Exclusion criteria included infants younger than 6 months of age (or weighting less than 5 kg), women in the first trimester of pregnancy, or severely ill individuals. All women of reproductive age (12–49 years of age) consenting to participate who reported not being pregnant or not knowing their pregnancy status were asked to take a urine-based pregnancy test (SD Bioline hcG, Standard Diagnostics, South Korea) at the time of the visit before taking DHAp. Women who tested positive were assumed to be in the first trimester and excluded. Women who refused a pregnancy test were warned of the potential detrimental effects of DHAp to the fetus and offered DHAp. More stringent exclusion criteria were applied during rounds 2 to 4 in response to a request from the National Ethics Committee and district health authorities (S1 Table).

A standardized electronic questionnaire using Open Data Kit (https://opendatakit.org/) was filled out for all participants, as well as for present or absent nonparticipating household residents, on household, sociodemographic, and malaria prevention information (S1 Appendix). A full dose of DHAp (once a day for three consecutive days) was provided to all consenting eligible individuals according to the age of the participant. Participants were advised to take the drug on an empty stomach and to not eat for at least one hour after taking the drug. Directly observed treatment was performed only on day 1. Doses 2 and 3 were left with the participants, who received instructions on how and when to take them. Adherence to DHAp was measured in a random subsample of MDA1 and MDA2 participants, who were visited one day after the last treatment dose (day 4) to measure the reported adherence (reported taking a correct full-treatment dose) and observed adherence (based on observation of the blister pack). An RDT (SD Bioline Ag *Pf*, Standard Diagnostics, South Korea) was performed for research purposes to all consenting MDA1 participants and to a random subsample of MDA2 participants prior to DHAp treatment, to estimate infection prevalence after the first IRS and first MDA rounds, respectively. S1 Table summarizes the procedures conducted during the MDA rounds.

The HF-based passive pharmacovigilance system of the MoH was reinforced in all HFs to monitor the adverse events (AEs) experienced by the individuals within 28 days since the intake of DHAp. The MDA team and the health professionals from all HFs were trained to report any AE from the MDA participants—irrespective of their severity or relationship to the drug. Serious adverse events (SAEs) were similarly detected and reported throughout all MDA rounds. A free phone line was active and available to anyone wishing to report any AE or SAE. Study physicians evaluated and reviewed all reported AEs within 28 days and guided clinical management, in addition to assessing their potential relationship to DHAp. Participants with SAEs were followed up until events resolved. Deaths occurring at home were investigated by a review of available medical records and by verbal autopsy (WHO 2012 instrument).

#### Reactive focal mass drug administration

A reactive focal mass drug administration (rfMDA) system was implemented in Magude during July 2017 and was fully operational by September of that year. All clinical malaria cases passively detected at the HFs or through the CHWs were considered “index cases” and DHAp was distributed to all eligible individuals in the index case household. The same procedures for informed consent, exclusion criteria, treatment, and data collection that were applied during MDA4 were followed. Only the first year of phase II was included in this manuscript.

#### Community-based cross-sectional surveys

Annual cross-sectional surveys were conducted at the end of the transmission season at baseline (May 2015), three months after MDA2 and MDA4 rounds (May 2016 and May 2017), and after one transmission season with rfMDA (May 2018). An age-stratified simple random sample of participants with oversampling of children under 15 years of age was conducted yearly using the census database as the sampling frame for children <6 months old, six months to two years, two to five years, five to 15 years, and adults 15 years old or older. The sample size was estimated every year to detect a 90% reduction in the age-specific prevalence with regard to the previous year during phase I, and to test for non-inferiority between 2017 and 2018 prevalence estimates (phase II). A standardized electronic questionnaire using REDCap was completed for every participant with basic sociodemographic, clinical, and vector control information (S2 Appendix). All consenting participants were finger-pricked to collect blood samples for malaria diagnosis by microscopy and RDT. Thick blood smears were screened for malaria parasites at the Centro de Investigação em Saúde de Manhiça (CISM) laboratory, using the Lambaréné method [17].

### Ethical considerations

All study protocols were approved by CISM’s institutional ethics committee, Hospital Clínic of Barcelona’s Ethics Committee, and the Mozambican Ministry of Health National Bioethics Committee. The study protocol to implement and evaluate the impact of MDAs and rfMDAs was also approved by the pharmaceutical department of the MoH of Mozambique and registered as Clinical Trial NCT02914145. Following meetings to inform about the project prior to its initiation, the district authorities of Magude agreed to the implementation of the project in writing. Written informed consent and assent (for 12 to 17 year olds) was sought from all individuals, or parents/guardians if participants were younger than 18 years old, who participated in each of the following studies before conducting any study procedures: population census, cross-sectional surveys, MDAs, and rfMDAs.

### Statistical analysis and study endpoints

The analysis of this study focused on the primary endpoints specified in each study protocol, as there was not a final prospective analysis plan. Crude study outcomes were reviewed on an annual basis, and the final analysis was conducted after June 2018, once all databases and laboratory results were available.

#### Intervention coverage

The coverage of the individual and combined interventions was measured during the baseline census; cross-sectional and MDA surveys and 95% confidence intervals (CIs) were calculated for the estimates obtained from the cross-sectional surveys. Weighted proportions were calculated to control for the overrepresentation of children in cross-sectional surveys when estimated for all ages or age groups that did not coincide with those used in the survey sampling. Care seeking after an episode of fever in the previous month was measured during the census and cross-sectional surveys. The coverage of visits to ANCs and IPTp was measured among the pregnant women in the second or third trimester found during MDAs 2, 3, and 4. Reported LLIN usage in the previous night and coverage of IRS in the previous 12 months were questioned at every survey. Reported IRS coverage estimates for the 2015 and 2016 MDA campaigns were obtained as the weighted mean of the proportion of households that reported having received IRS during the previous 12 months. This information was obtained from participating households during the MDA rounds (rounds 1/2 combined [2015] and 3/4 combined [2016]). The coverage of the 2017 IRS campaign was obtained from households that participated in the parasite survey in May of 2018. The operational IRS coverage was reported by GBM as the number of eligible structures sprayed out of the target structures.

The effective MDA coverage was estimated as the number of individuals treated divided by the population at risk (PAR), defined as the number of individuals reported or identified to be in Magude during the MDA visit, regardless of whether they were present or absent at the time of the visit and whether or not they had been previously censed [18]. The operational MDA coverage was defined as the number of individuals treated divided by the number of individuals present in the household at the time of the MDA visit. The effective and operational coverage of each MDA round was calculated for all ages as well as for individuals younger and older than five. The distributions of the sociodemographic characteristics of the participants of each MDA round were calculated separately for those who were treated, excluded (but present during the MDA), or missed at the time of the visit (but were identified during the census and contributed to the PAR). For the rfMDA, the index-case reaction coverage was measured as the number of index cases for which rfMDA was conducted at their households within 72 hours of detection divided by all index cases eligible for follow-up.

The reported combined coverage of vector control interventions (living in a household sprayed in the previous 12 months and/or sleeping under a net the previous night, or none) was measured during the baseline census and cross-sectionals of 2015, 2016, and 2018. The cross-sectional of 2017 measured the combination of reported MDA and vector control coverage. During the MDAs, the coverage of any combination of vector control was measured among those who were treated (thus participated in the MDA) and those who were excluded from the MDA but were present in the household.

#### Community-based malaria infection prevalence

Annual community-based prevalence estimates of *P*. *falciparum* infection by microscopy and RDT and geometric mean parasite densities (GMPDs) measured with microscopy were calculated among those for whom results were available. Prevalence was estimated by age group (<6 months olds, six months to two years, two to five years, five to 15 years, and adults ≥15 years old) and using the data collected from the community surveys, MDA1 and MDA2. The proportion of afebrile infections was also calculated as RDT-positive individuals with an axillary temperature <37.5°C and who did not report having a fever in the previous 24 hours. Proportions and GMPDs with their 95% CIs were estimated and compared using Z-tests and Wilcoxon rank-sum tests, respectively. Analyses were done using Stata version 14 (Stata Corp, College Station, TX).

#### Passively detected malaria case incidence and inpatient admissions and deaths

Age-specific annual malaria incidence risks of passively detected cases by RDT or microscopy were estimated for each transmission year (July 1 of one year to June 30 of the following year). Routine data were complete for all months of the study period. Risks were calculated at the district level as the number of malaria cases detected at HFs and by CHWs in Magude reported to the RRS divided by the total or age-specific population of Magude for that year. Population denominators were obtained from projected age-stratified population estimates from the Mozambican National Institute of Health. Monthly medians of the test positivity rate (TPR) were calculated per study phase as the number of positive cases among those tested. The inpatient malaria data from the Magude Sede health center were used to depict trends in the number of all-cause and malaria-associated admissions and deaths. The distribution of the passively detected AEs and SAEs were calculated per MDA round.

#### Climatic data

Enhanced vegetation index (EVI) (MOD13A3) estimates were retrieved using the rts R package [19] from moderate resolution imaging spectroradiometer (MODIS) satellite data [19]. Rainfall data were obtained from the Climate Hazards Group InfraRed Precipitation with Station data (CHIRPS) [20]. Data from every raster file per month were extracted for every household in Magude and transformed to weekly data to be used as covariates in the interrupted time series (ITS) analysis. Daily average temperature estimates were obtained from the National Oceanic and Atmospheric Administration (NOAA) collected by the Maputo Weather Station (station ID 673410) (S1 Fig).

#### Impact evaluation

An ITS analysis was used to estimate the impact of the combination of all interventions deployed in each phase. The model initially included a random effects term at the health-facility catchment area (HFCA). However, this term was later removed given the overreporting of cases in the HF of Magude Sede that came from other HFCAs. As a result, the final model used the aggregated district-level weekly number of malaria cases reported to the RRS (positive by RDT or microscopy, reported by HFs or CHWs) as the dependent variable. A generalized estimating equations (GEEs) model with Poisson distribution and an autoregressive correlation structure matrix was used. To estimate the impact of phase I (interventions deployed between August 2015 and 2017) and phase II (September 2017 to June 2018), two interruptions were included in the ITS model coinciding with the first IRS campaign of each phase: one at the beginning of phase I, in August 2015, and a second one at the beginning of phase II, in September 2017. For each interruption, a change in level (immediate phase effect) and a trend term (interventions’ effect over time) were included in the model [21], with the assumption that the combination of IRS followed by MDA would have a strong immediate impact on transmission, as observed in previous trials in Africa [22,23]. The ITS model assumed the following equation:

$$Y_{t}=\beta_{0}+\beta_{1}T_{t}+\beta_{2}Int_{1}+\beta_{3}Int_{1}*T_{t}+\beta_{4}Int_{2}+\beta_{5}Int_{2}*T_{t}+\Sigma \beta_{j}Covariates+\varepsilon_{t}$$

Wre Y_t_ is the number of weekly (t) cases of malaria aggregated at district level; T_t_ is the number of weeks since the beginning of the time series (first week of October 2013); Int_1_ is a dummy variable representing phase I intervention (pre-intervention period as 0, post-intervention period as 1); Int_1_*T_t_ is an interaction term (phase I); Int_2_ is a dummy variable representing phase II interventions; and Int_2_*T_t_ is an interaction term (phase II). β_0_ represents the starting level of malaria cases; β_1_ is the slope of malaria cases until the introduction of phase I interventions; β_2_ represents the change in the level of cases that occurred in the period immediately following the introduction of phase I interventions (i.e., the immediate effect of phase I interventions); β_3_ is the difference between preintervention and phase I slopes (effect over time of phase I interventions compared with preintervention); β_4_ represents the change in the level of cases that occurred in the period immediately following the introduction of the phase II interventions (immediate effect of phase II interventions); β_5_ is the difference between phase I and phase II slopes (effect over time of phase II interventions compared with phase I), and ε_t_ is the error term (S3 Appendix).

The following covariates were considered to be a priori associated with the dependent variable and included in the model: mean rainfall with a lag of one month, average temperature with a lag of one month; mean EVI with a lag of one and two months, the weekly number of passively detected non-malaria cases (to control for variations in care-seeking or reporting rates), and the bednets per capita distributed by the NMCP with a lag of 1 month. The bednets variable included a linear integrity decay function assuming 100% integrity within the first six months after the 2014 distribution, a 20% reduction in the number of fully functioning nets after two years (based on a net integrity assessment conducted in Magude), and an extension of this linear decay until the 2017 net distribution. The covariates’ lags and the autoregressive correlation structure matrix were selected using the quasi-likelihood under the independence model criterion (QIC) [24].

The final model was used to estimate a counterfactual of the weekly number of malaria cases predicted to have occurred in the district during the intervention periods, had the interventions of the Magude project not been implemented. The weekly number of averted malaria cases was then estimated as the difference between the expected number of cases in the counterfactual and the observed number of cases. This analysis was performed using statistical software R version 3.4 [25].

## Results

### Intervention coverage

Between 58.4% and 76.6% of individuals who reported having a fever in the preceding 30 days during the cross-sectional surveys sought healthcare at a HF. Between 69% and 75% of pregnant women in the second or third trimester found during the MDAs 2–4 reported having attended at least one ANC visit. More than 90% of the women who attended the ANC reported receiving at least one dose of IPTp (Table 1).

**Table 1:** Summary of intervention coverage (%) measured throughout the Magude project through population-level surveys conducted during the baseline census, the four rounds of MDA, or through age-stratified cross-sectional surveys conducted at the end of the malaria transmission season. Coverage estimates obtained at the individual level from the cross-sectional surveys were weighted to control for the age distribution of the sample.

INTERVENTIONS		BASELINE (%)		PHASE I (%)						PHASE II (%)	
Source of data		Census	Cross-sectional survey^1^	MDA1	MDA2	Cross-sectional survey^1^	MDA3	MDA4	Cross-sectional survey^1^^,^^2^	rfMDA	Cross-sectional survey^1^
*Study period*		Jan–Jun 15	May 15	Nov 15	Jan 16	May 16	Dec 16	Feb 17	May 17	Jul 17–Jun 18	May 18
*Sample size*		*48*,*448*	*1*,*035*	*43*,*439*	*37*,*675*	*1*,*657*	*39*,*759*	*39*,*748*	*3*,*865*	* *	*3*,*354*
**INDIVIDUAL INTERVENTIONS**										
Sought care for fever in previous 30 days			40.9			64.4			72.2		70.6
ANCs (at least one visit)					69		70.6	75			
IPTp (at least one dose)					95.7		90.4	92			
LLIN usage		25.4	40.9	67.9	76.3	64.4	67.8	70.3	72.2		70.6
IRS: Effective coverage^+^		52.2	61.8	77.8	83	84.3	84.5	89.7	76.9		73.5
Community engagement messages^+^				94.8			95.8				
MDAs: Operational				90.6	75.4		85.4	83.9			
MDAs: Effective				72.3	58		66.6	64.8	87.5		
MDA Adherence: Reported^§^				83	83						
MDA Adherence: Observed^§^				73	62						
rfMDA Index case coverage										79.3	
**COMBINATION OF INTERVENTIONS**										
***Vector control***	***MDAs***^***#***^										
None	No		22.4	0.6	0.6	7.6	0.6	0.4	1.4		10
	Yes			6.0	3.0		4.4	2.7	2.7		
LLINs	No		36.7	0.8	1.1	27.7	0.9	0.7	3.6		20
	Yes			9.3	6.1		5.4	3.9	5.2		
IRS	No		13.2	1.6	2.9	7.1	3.0	3.1	8		15.5
	Yes			21.0	15.7		24.1	23.2	11.1		
IRS + LLINs	No		27.8	3.7	10.2	57.6	6.8	8.8	19		54.4
	Yes			57.0	60.4		54.8	57.1	49		

^1^Weighted estimates obtained from an age-stratified subsample of the population.

^2^The only cross-sectional survey where the reported coverage of MDAs 3 and 4 was measured.

^+^Household-level estimates.

^§^Estimates obtained from a subsample of MDA1 and 2 participants.

^#^Intervention coverage during the MDAs was measured among those who participated in the MDA campaigns and were treated (MDA = Yes) or excluded (MDA = No). The cross-sectional survey of 2017 evaluated the reported coverage of all interventions.

Abbreviations: ANC, antenatal clinic; IPTp, intermittent preventive treatment for pregnant women; IRS, indoor residual spraying; LLIN, long-lasting insecticidal net; MDA, mass drug administration; rfMDA, reactive focal mass drug administration

The usage of LLINs reported during the baseline population census (25.4%) and cross-sectional survey of 2015 (40.9%) increased to 64.4%, 72.2%, and 70.6% in the 2016, 2017, and 2018 surveys, respectively (Table 1). Children under 5 years reported using a net significantly more than those older than five in 2017 and 2018 (S2 Table). The reported coverage of the IRS campaigns of 2015, 2016, and 2017 was 80.2%, 87.1%, and 73.5% (95% CI 71.3–75.6), respectively. The operational IRS coverage was 94.4%, 94%, and 98.4% in 2015, 2016, and 2017, respectively (Table 1).

Among household heads that participated in MDA rounds 1 and 3, 94.5% and 95.8% reported having received messages from the community engagement activities prior to the MDA campaigns. The effective MDA coverage was 72.3% and 58% in the first and second round, respectively, and 66.6% and 64.8% in the third and fourth round, respectively (Table 1). Coverage was higher in children under five years (>70%) compared with those older than five (50%–70%) in all MDA rounds (S2A Fig and S2B Fig). Among MDA2 and MDA4 participants, 51.7% and 58.5% had also been treated in MDA1 and MDA3, respectively. The adherence assessment revealed that 83% of participants in MDA1 and MDA2 reported taking the correct dose of the medication for the three-day period. The percentage of participants that showed an empty blister pack on day 4 decreased significantly from 73% in MDA1 to 62% in MDA2. Since September 2017, rfMDA was conducted for a median of 79.3% of the index cases detected (Table 1).

The spatial distribution of all interventions (MDAs, IRS, and LLINs) was homogeneous throughout the district (S3 Fig and S4 Fig). However, there was a higher proportion of individuals older than 15 years of age who were either excluded or missed during the MDA campaigns. Women, individuals with no formal education, and farmers or fishermen were more commonly excluded from DHAp treatment, while there was a slight underrepresentation of men present during the MDA visits (S3 Table). The proportion of individuals who reported not being covered by any vector control intervention decreased from 22% at baseline, to 7.6% and 4.1% (phase I), and to 10% in 2018. During the cross-sectional survey of 2017, more than 65% of the surveyed population reported having been covered by MDA and at least one form of vector control (Table 1).

### Infection prevalence and cinical incidence

**Fig 2:**
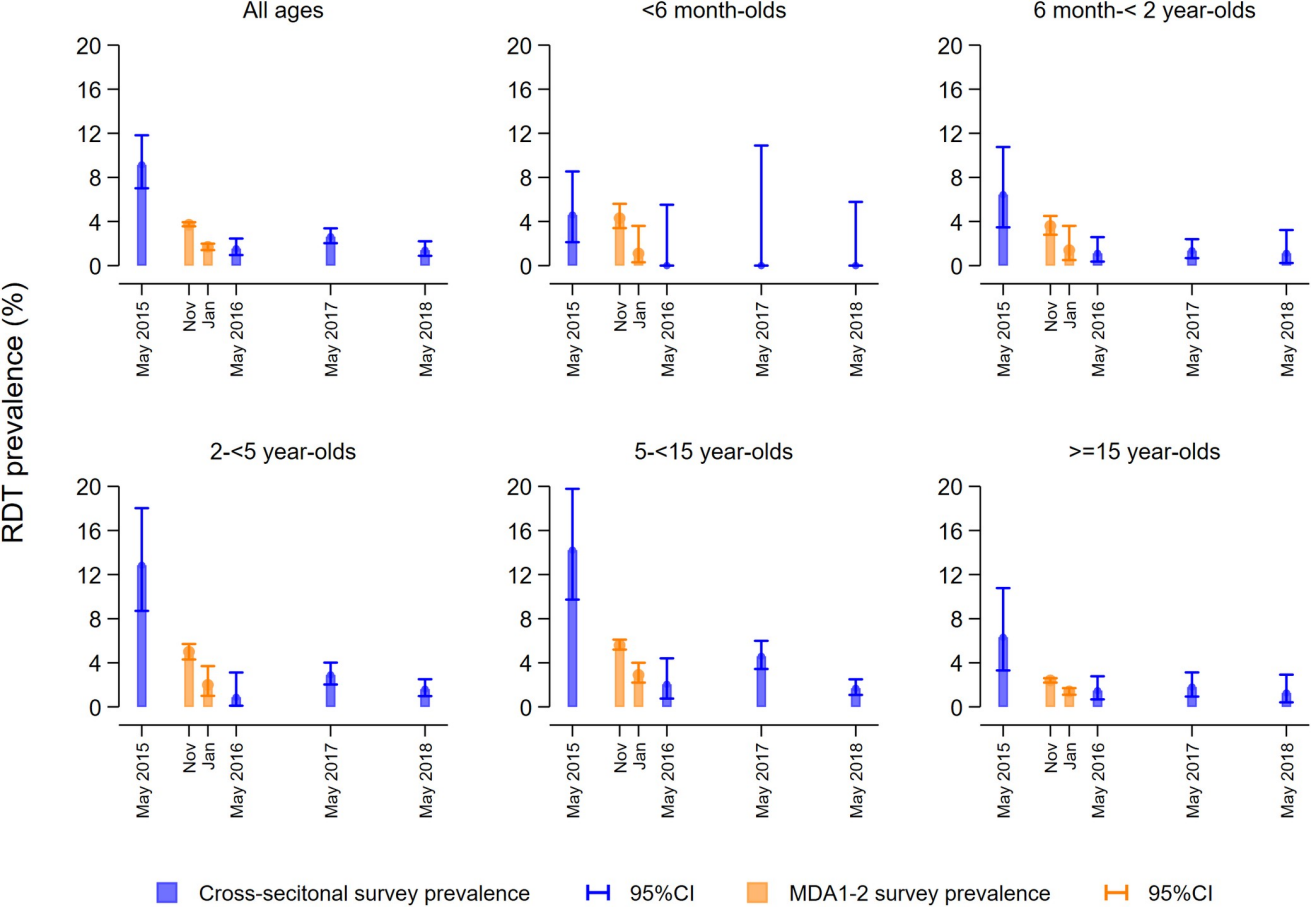
All-age and age-specific malaria infection prevalence estimates measured by RDT before (May 2015) and during the Magude project (2016–2018). CI, confidence interval; MDA, mass drug administration; RDT, rapid diagnostic test.

All-age weighted baseline prevalence by RDT in May 2015 was 9.1% (95% CI 7.0–11.8) and dropped to 3.7% (95% CI 3.6–3.9) in MDA1, 1.7% (95% CI 1.4–2.0) in MDA2 and 1.5% (95% CI 1.0–2.5) in May 2016. In May 2017, the RDT-prevalence was 2.6% (95% CI 2.0–3.4), representing a 71.3% (95% CI 71.1–71.4, *p* < 0.001) reduction relative to baseline. The survey conducted in May 2018 showed a prevalence of 1.4% (95% CI 0.9–2.2) by RDT and an overall reduction of 84.7% (95% CI 81.4–87.4, *p* < 0.001) compared with baseline (Fig 2 and Table 2). RDT prevalence estimates varied significantly across age groups throughout the project. Among infants <6 months of age, RDT prevalence was 4.6% (95% CI 2.1–8.5) at baseline and 0% thereafter (Fig 2). The GMPDs measured by microscopy did not significantly change across years. The proportion of RDT-positive individuals who were afebrile [24] ranged from 63.3% to 71.3% throughout the project (S2 Table).

**Table 2:** Observed reduction in study outcomes per project phase. Reductions observed in age-specific microscopy and RDT prevalence and incidence, and the estimated proportion of cases averted during phase I and phase II of the project.

*Indicator*	*Age group*	Phase I			Phase II			Phases I and II
		August 2015 to June 2017			July 2017 to June 2018			August 2015 to June 2018
		*Baseline*^*1*^	*Endpoint*^*2*^	*Percent reduction*	*Baseline*^*2*^	*Endpoint*^*3*^	*Percent reduction*	Percent reduction*
**Infection prevalence by microscopy**	<6 months	4.2 (1.8–8.1)	0 (0–5.7)	100	0 (0–5.7)	0 (0–6.0)	NA	100
	6 months–2 years	5.1 (2.5–9.1)	0.5 (0.2–1.4)	89.3 (93.9–84.7)	0.5 (0.2–1.4)	0.4 (0.0–2.1)	29.6 (93.3 to −52.5)	92.5 (99.6–76.7)
	2–<5 years	8.8 (5.4–13.5)	2.9 (2.0–4.0)	67.2 (62.4–70.2)	2.9 (2.0–4.0)	0.9 (0.4–1.6)	69.7 (79.3–59.6)	90.0 (92.2–88.0)
	5–<15 years	10.4 (6.6–15.5)	1.6 (0.9–2.6)	84.5 (86.0–83.2)	1.6 (0.9–2.6)	1.3 (0.7–2.0)	22.4 (20.7–23.5)	88.0 (88.9–87.1)
	≥15 years	5.4 (2.6–9.7)	0.3 (0.0–1.2)	93.7 (98.5–87.5)	0.3 (0.0–1.2)	1.1 (0.3–2.7)	−208.8 (−625.0 to −120.7)	80.5 (88.9–72.4)
	**All ages**	**7.1 (5.2–9.6)**	**0.8 (0.5–1.2)**	**89.3 (90.8–87.6)**	**0.8 (0.5–1.2)**	**1.0 (0.6–1.8)**	**−35.5 (−20.8 to −52.9)**	**85.5 (88.8–81.1)**
**Infection prevalence by RDT**	<6 months	4.6 (2.1–8.5)	0	100	0	0	NA	100
	6 months–2 years	4.6 (2.1–8.5)	1.4 (0.7–2.4)	70.6 (68.4–71.9)	1.4 (0.7–2.4)	1.1 (0.2–3.2)	17.0 (65.7 to −34.2)	75.6 (89.2–62.3)
	2–<5 years	12.8 (8.7–18.0)	0.9 (0.5–1.7)	92.8 (94.8–90.6)	0.9 (0.5–1.7)	1.6 (1.0–2.5)	−73.1 (−115.6 to −47.6)	87.5 (88.9–86.1)
	5–<15 years	14.2 (9.7–19.8)	4.6 (3.4–6.0)	67.7 (64.6–69.7)	4.6 (3.4–6.0)	1.7 (1.1–2.5)	63.2 (68.6–58.3)	88.1 (88.9–87.4)
	≥15 years	6.3 (3.3–10.8)	1.8 (0.9–3.1)	71.5 (71.9–70.9)	1.8 (0.9–3.1)	1.3 (0.4–2.9)	30.0 (55.9–6.7)	80.1 (87.6–72.9)
	**All ages**	**9.1 (7.0–11.8)**	**2.6 (2.0–3.4)**	**71.3 (71.1–71.4)**	**2.6 (2.0–3.4)**	**1.4 (0.9–2.2)**	**46.6 (56.7–34.9)**	**84.7 (87.5–81.4)**
**Malaria case incidence**	<5 years	298 per 1,000	83 per 1,000	72.1	83 per 1,000	69 per 1,000	16.9	76.8
	≥5 years	58 per 1,000	16 per 1,000	72.4	16 per 1,000	13 per 1,000	18.8	77.6
	**All ages**	**195 per 1,000**	**75 per 1,000**	**61.5**	**75 per 1,000**	**67 per 1,000**	**10.7**	**65.6**
**Estimated percent of cases averted**	**All ages**	** **	** **	**75.6**	** **	** **	**79.1**	**76.7**

^1^May 2015 for prevalence, and the transmission year of July 2015 to June 2016 for incidence.

^2^May 2017 for prevalence, and the transmission year of July 2016 to June 2017 for incidence.

^3^May 2018 for prevalence, and the transmission year of July 2017 to June 2018 for incidence.

*Comparing Phase I baseline^1^ with Phase II endpoint^3^_._

Abbreviation: RDT, rapid diagnostic test

**Fig 3:**
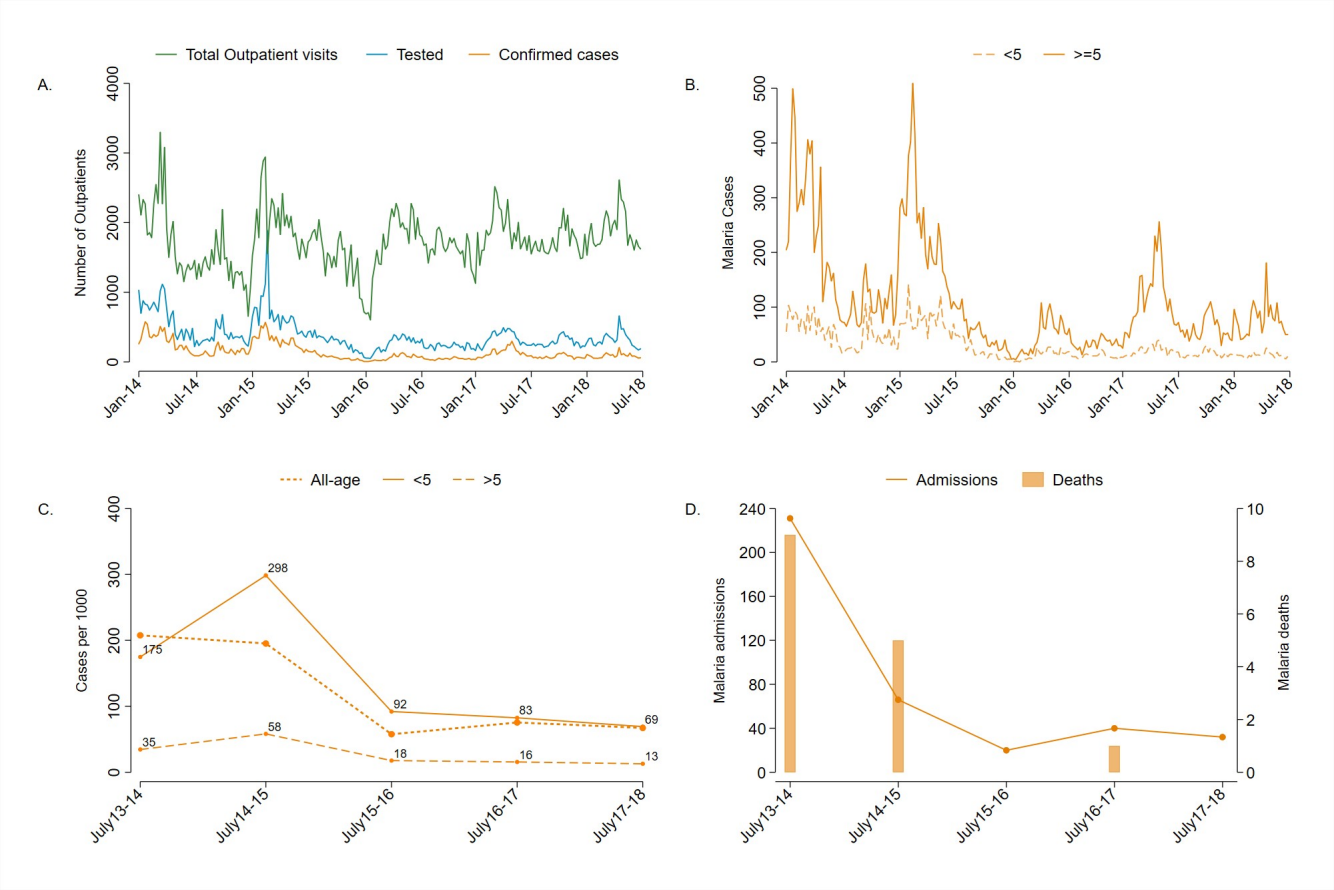
Routine outpatient data collected through the RRS of Magude. (A) Weekly number of outpatients who attended any HF of Magude (dotted line), the diagnostics performed (dashed line) and cases confirmed (solid line). (B) Weekly number of cases in children under 5 years and individuals five years old and older. (C) All-age, <5-year-old, and ≥5-year-old annual incidence rates reported by the HFs and CHWs through the period before (July 2013–2015) and during the malaria elimination project (July 2015–2018). (D) Number of malaria-associated inpatient admissions and deaths. CHW, community health worker; HF, health facility; RRS, rapid reporting system.

Prior to the project, there were 12,482 malaria cases reported in the Magude district during the transmission year of July 2013–June 2014 and 11,923 between July 2014 and June 2015. The first and second transmission-years of the project (phase I) observed 3,578 cases and 4,749 cases, respectively. An additional 4,289 cases were reported during the transmission year of July 2017–June 2018 (phase II). Consequently, the all-age annual incidence of malaria cases at baseline was 195 per 1,000 (2014–2015 transmission year) and declined to 75 cases per 1,000 during the 2016–2017 season (61.5% reduction). The 2017–2018 transmission year had an incidence of 67 cases per 1,000, a reduction of 65.6% compared to baseline, and of 10.7% since the discontinuation of MDAs. Similar trends in incidence were observed in <5 and ≥5-year-olds (Fig 3A–3C). The median of the monthly TPRs at baseline was 45% (IQR 31.3 to 58.7) and declined to a median TPR of 25.7% (IQR 9.3 to 42.1) during phase I and 27.1% (IQR 18.3 to 35.8) during phase II. A decreasing trend in all-cause inpatient admissions and deaths started prior to phase I activities to 66 malaria-associated admissions and 5 deaths between July 2014 and June 2015, according to admitting clinicians. The three transmission seasons covered by the project recorded 20, 40, and 32 malaria admissions, respectively, and only one malaria death in 2016 (Fig 3D).

### Estimated impact of interventions and number of cases averted

**Fig 4:**
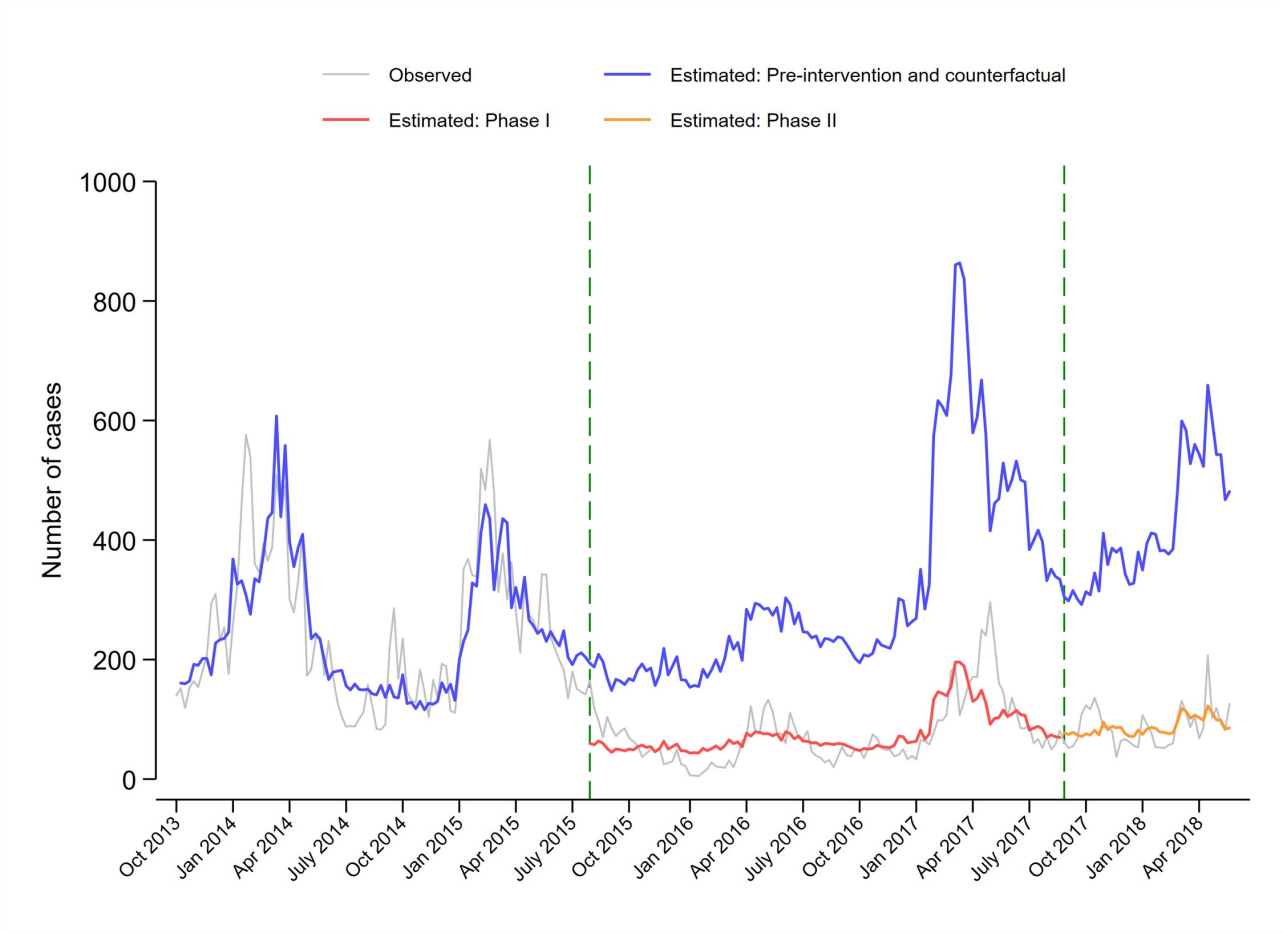
ITS regression model estimates of the weekly number of passively detected malaria cases in Magude before (October 2013 to July 2015) and after the intervention during phase I (August 2015–2017) and phase II (September 2018 to June 2018), and the estimated cases that would have happened since the first phase of the Magude project, had this not taken place (counterfactual). ITS, interrupted time series.

The ITS model estimated that there was a significant level reduction (expß_2_ = 0.309, 95% CI 0.225–0.425, *p* < 0.001) in August 2015, with a drop of 69.1% in the number of cases at the time of the intervention. The weekly trend of malaria cases during the implementation of phase I interventions also decreased relative to the pre-intervention trend (expß_3_ = 0.996, 95% CI 0.991–1.002, *p* = 0.202). There was no significant-level change in September 2017 (expß_4_ = 1.224, 95% CI 0.889–1.703, *p* = 0.231) or change in the trend during phase II (expß_5_ = 0.994, 95% CI 0.981–1.008, *p* = 0.402) relative to the phase I trend (S4 Table). Of the 50,005 estimated cases expected, 38,369 cases (95% CI 38,184–38,555) were averted (76.7% of expected cases, 95% CI 76.4–77.1) between August 2015 and June 2018, of which 25,472 (95% CI 25,312–25,627) cases were averted during phase I (75.6%, 95% CI 75.1–76.0), and 12,897 (95% CI 12,796–12,999) cases were further averted during phase II (79.1%, 95% CI 78.5–79.8) (Fig 4).

### Safety outcomes of the MDA rounds

There were 109, 74, 77, and 17 AEs recorded through the passive pharmacovigilance system during the four MDA rounds, respectively. The most common AEs during the entire MDA campaigns included headache (0.052%) vomiting (0.034%), asthenia (0.022%), fever (0.019%), and abdominal pain and dizziness (0.013% each) (S5 Table). None of the AEs progressed to SAEs.

Two deaths were also captured through the pharmacovigilance system during the prespecified time at risk (28 days) post each MDA dose. The first death took place on day 2 of the first MDA round. This was a sudden unexplained death of an otherwise healthy 16-year-old female who self-administered the second dose of DHAp (3 tablets of the 320/40 mg formulation, consistent with a daily target dose of 4 mg/kg/day dihydroartemisinin and 18 mg/kg/day piperaquine) approximately 20 minutes after a meal of rice, cooked salad, and bread, in spite of the team’s recommendation for taking the drug on an empty stomach. She subsequently complained of palpitations, collapsed, and died on her way to the hospital. A detailed investigation was triggered upon initial notification (within 24 hours of the event), which consisted of a verbal autopsy to a close member of her family (her stepmother), and the review of all her study and hospital records. This investigation revealed that she had no history of previous hospital admissions or any other medical conditions, and no past or concomitant intake of any other medication. Both the malaria RDT and pregnancy test performed at her enrolment the day before were negative, and no complaints had been noted after the first dose of DHAp taken the preceding day. No autopsy or ECG could be performed. This death was considered consistent with a sudden cardiac event and potentially related to the drug by the study team, as well as by a group of experts of the WHO Evidence Review Group on the Cardiotoxicity of Antimalarials [26].

The second death of a 43-year-old female took place 6 days after taking DHAp during MDA3. This individual was under antiretroviral treatment and had no history of cardiovascular disease. She had been admitted into a hospital with abdominal pain and distention and was diagnosed with intestinal occlusion. This event was deemed not to be related to the intake of the drug by the study team.

## Discussion

A before-after study was conducted in the district of Magude to evaluate the feasibility of malaria elimination in sub-Saharan Africa using a comprehensive mix of interventions recommended by the GTS. Building on an enhanced surveillance system and community engagement program, two rounds of MDA per year over two years (phase I) followed by one year of rfMDA (phase II) were deployed together with annual IRS, programmatically distributed LLINs, and standard case management. The primary endpoints of the study were measured using weekly routine malaria data of cases, admissions and deaths, and cross-sectional surveys to estimate all-age and age-specific *P*. *falciparum* prevalence. The impact of each phase of the project was estimated using an ITS analysis.

During the implementation of the Magude project (phase I and II), the parasite prevalence and annual incidence of malaria cases dropped by 84.7% and 65.6%, respectively. Both these indicators speak to a very substantial reduction in transmission intensity. Furthermore, the fact that no infections were detected among the infants less than 6 months of age in cross-sectional surveys after the first round of IRS and MDAs implies a drastic reduction of the force of infection [27], and suggests that the mix of interventions was close to interrupting transmission. After controlling for programmatic activities and changes in environmental factors, we estimated that 38,369 cases were averted (76.7% of expected cases) in a population of approximately 50,000 over a three-year period. The observation of only one malaria-associated inpatient death since the project started suggests a reduction in malaria mortality associated with the drop of transmission. Overall, these results represent a failure to achieve interruption of *P*. *falciparum* transmission, but a large success in reducing the burden due to this lethal parasite.

While the study design did not allow measuring intervention-specific effects, the parasite surveys performed during MDA1 (i.e., after the 2015 IRS round) and MDA2 (i.e., 6 weeks after the first MDA round) indicate a substantial reduction in prevalence of infection after the implementation of IRS, which was followed by a further drop observed after MDA1. In other words, well-conducted IRS in the presence of LLINS, and in an area with significant pyrethroid resistance [13], provided an increased level of protection. The reduction in parasite prevalence following MDA1 further argues on the additive effect of interventions aiming to reduce parasite biomass on top of vector control. Furthermore, switching from population-wide MDA to rfMDA while sustaining IRS, and with an additional mass LLIN distribution, was not associated with reduced incidence or reversal of the gains for a year since the suspension of MDAs. There have been a number of previous attempts in different settings in Africa aiming to interrupt transmission, from the elimination attempts in the 1960s and 1970s, such as the Garki or the Kankiya project, to the recent trials conducted in Zambia or Comoros [23,28,29]. Allowing for significant differences in designs, interventions, and follow-up times, they all share a common result also reported in this study, which is that they all failed to interrupt malaria transmission [22].

The question remains as to why we are falling short of interrupting transmission, and what strategies are required to achieve this goal [18,30,31]. First, the coverage of the different interventions may have been insufficient, resulting in a proportion of the population hosting a significant parasite reservoir [18,32,33]. Despite reaching a relatively high coverage, IRS implementation was affected by absenteeism or lack of availability of household heads, or refusals to accept the intervention due to reasons such as the perceived smell or coloring of the walls due the insecticide. The coverage and adherence to MDA and rfMDA was more challenging and affected by several factors: (a) missing individuals during household visits, who are difficult to identify and account for, especially if they were not censed; (b) the application of stringent exclusion criteria as a result of the SAE observed in MDA1, and in the context of a high prevalence of HIV infections in the population, with more contraindications to antibiotic or antifungal treatments; (c) incomplete adherence rates to the full treatment course potentially associated with the perception that treatment is not needed or to a fear of AEs. Despite all these factors, evidence from the cross-sectional survey of 2017 suggests that by the end of phase I, only 1.4% of the population was not covered by any of the interventions, and more than 65% of the population was covered by the MDAs and at least one form of vector control. Additionally, the individual-level data collected during the MDA surveys revealed that the second and fourth rounds of MDA covered a large number of individuals (around 45%) who were not covered by the first or the third rounds. This could have been due to population mobility, the timing of each round (as MDAs coincided with the beginning and end of the holidays), or challenges in matching the information for the same person through different rounds. Overall, this suggests that conducting more than one round of MDA per year is needed to increase the population coverage of the intervention. It also reveals that the majority of the population that participated in the annual MDA campaigns only received one of the two rounds and was thus under chemoprevention with DHAp for 1–2 months instead of 2–4 months [34], thus increasing the potential for exposure to local or imported infections [15,35,36].

Second, population movement may have led to a continuous influx of parasites from neighboring endemic areas, which could have sustained transmission in the face of significant receptivity. Third, recognizing that all the tools and intervention strategies used are imperfect, significant residual transmission may have also contributed to sustain transmission. Finally, the duration of the efforts to rapidly reduce transmission to zero cases (phase I) may have been too short. Indeed, the expectation that with imperfect tools and imperfect coverage we may have been able to interrupt *P*. *falciparum* transmission in Africa’s mainland through a time limited effort of two years may have been simply overoptimistic.

Thus, future attempts to interrupt transmission with MDA should consider two components—the first, optimizing uptake and adherence of the interventions. In the case of MDA, this intervention may require the implementation of several consecutive rounds of MDA per year to increase coverage [18] and ensure that individuals are treated at least in one, and ideally in more than one, round to extend the chemoprevention period. In Magude, the increase in LLIN usage observed throughout the project was very likely associated with the community engagement activities implemented since its onset. While community engagement messages also focused on increasing access to care in Magude, this was suboptimal, as between 30% and 40% of individuals with fever reported not seeking care in Magude. This can probably be explained by the long distances between some areas and the HFs or by the perception of low-quality services provided by the health staff. Therefore, similar projects in the future should consider performing a pre-assessment of the distribution of their CHWs, as well as more emphasis on healthcare worker’s training and supervision. This is particularly relevant in endemic contexts where immunity does not wane as a result of drastic transmission reductions, and subpatent and potentially transmissible infections will remain in the immune population without becoming symptomatic [37,38], which was evident from the high prevalence of afebrile infections detected throughout the project. Second, efforts to tackle residual transmission [39] and importation of infections [35,36] should also be considered, but strategies to do so remain unclear and research in these areas is key.

This study had a number of strengths and limitations. The primary outcome of the study depended on the quality of the routine data collected during the project, which the DQAs conducted as part of the strategy to strengthen the malaria surveillance system in the south of the country [9] revealed to be highly complete, timely, and accurate. We chose to use routine data, given that this is the main source of information that countries have available to evaluate the impact of interventions in programmatic mode, which is what this project intended to reproduce. Data from the cross-sectionals and MDA rounds may be subject to data collection errors, misinforming, or reporting bias. However, the consistency of the results obtained through the different surveys indicates that the information collected was sufficiently robust. Prevalence estimates measured in this study are likely to be underestimates of the true prevalence of infection in the community, which includes subpatent infections that were probably missed by microscopy and RDTs [37]. In spite of this, these diagnostics are also the most common forms of diagnostics accessible to malaria control programs, unless more sensitive field-deployable tools become available. The impact evaluation of the study was limited by the short pre-intervention period included in the ITS analysis, which was used to estimate the post-intervention counterfactuals in the absence of the project interventions. This analysis was also affected by the irregular rainfall patterns experienced in the district during the project, mainly affected by the El Niño and La Niña events that lead to an uncommonly dry season during the first year of phase I (2015–2016) and to heavy rainfall during the second year of phase I (2016–2017).

Overall, the findings obtained through the Magude project suggest that MDA could be considered a chemoprevention tool useful to accelerate towards malaria elimination in areas of low to moderate transmission intensity in Africa, where standard case management, vector control, and enhanced surveillance are in place, as reported in other low to moderate transmission areas of Sub-Saharan Africa [22,28,29,40,41]. The use of MDAs has at times been controversial, given the questions posed around its safety when distributing a drug with potential side effects to a large number of healthy individuals [42]. Here, we show that MDAs were generally safe, but one death potentially associated with DHAp was also detected. Nonetheless, the cardiotoxicity of the drug has been evaluated by a WHO committee that has concluded that DHAp falls within the acceptable ranges of safety [43]. As a result, the decision on whether to implement strategies that aim to drastically reduce transmission in African countries will be mainly driven by their cost-effectiveness and sustainability, which we explore in a separate publication [44].

## Conclusion

Results from this project indicate that the implementation of an optimized package of interventions in areas of low to moderate transmission in sub-Saharan Africa can achieve a significant reduction in burden but ultimately failed to interrupt malaria transmission. In spite of this, the Magude project revealed that an intensive implementation of currently available tools recommended by WHO can achieve major reductions in malaria transmission and burden of disease. All in all, this suggests that in low to moderate endemic areas of sub-Saharan Africa, malaria elimination is likely to be a long-term goal that will require sustained funding and national ownership for success. Nevertheless, at a time when the global fight against malaria is plateauing at an unacceptably high level, with over 200 million cases and an excess of 400 thousand deaths, a large majority of them among African children and women, our global public health priority has to focus on reducing disease and death, particularly among the most vulnerable populations. This should be viewed as an essential step of the pathway towards elimination.

## Supporting information

S1 Checklist Strengthening the Reporting of Observational Studies in Epidemiology (STROBE) checklist. (DOCX)

S1 Fig Monthly rainfall and EVI in Magude District and mean temperature in Maputo city (October 2013–June 2018). Rainfall raster data were obtained from the CHIRPS. EVI (MOD13A3) raster files were retrieved from MODIS satellite data. Data from every raster file per month were extracted for every household in Magude. Daily average temperature estimates were obtained from the NOAA collected by the Maputo Weather Station (station ID 673410) and aggregated monthly. CHIRPS, Climate Hazards Group InfraRed Precipitation with Station data; EVI, enhanced vegetation index; MODIS, moderate resolution imaging spectroradiometer; NOAA, National Oceanic and Atmospheric Administration.
(TIF)

S2 Fig Coverage estimates of four MDA rounds and rfMDA in Magude. (A) Age-stratified coverage cascade of the population groups considered for the estimation of the effective and operational coverage of each MDA round (blue, PAR; green, present at the time of the MDA visit; orange, eligible for DHAp treatment; red, treated with DHAp; gray, missing information for age in any category). (B) Effective and operational coverage for <5 and ≥5-year-olds per MDA round. (C) Percentage of index cases detected at the HF/CHW for which an rfMDA response at the index case household was conducted. CHW, community health worker; DHAp, dihydroartemisinin-piperaquine; rfMDA, reactive focal mass drug administration; MDA, mass drug administration; PAR, population at risk; rfMDA, reactive focal mass drug administration.
(TIF)

S3 Fig **Maps of households covered by MDA1 (A), MDA2 (B), MDA3 (C), and MDA4 (D).** Each map presents the households identified during the census of 2015 and 2016 that were not visited by the MDAs (red), households visited by the MDAs where no members were treated (orange), and households where at least one member was treated (blue). MDA, mass drug administration.
(TIF)

S4 Fig **Maps of households that reported owning at least one LLIN one and two years after the LLIN distribution that took place in May of 2014 (A) and (B), and households that reported receiving IRS in the previous 12 months (C) and (D).** Information on LLIN ownership was obtained from all the households censed during the census conducted in 2015 and 2016. Information on IRS was obtained from all households that participated in the MDA campaigns that took place immediately after the IRS campaign (i.e., MDA1 to evaluate the coverage of IRS in 2015, and MDA3 for the IRS of 2016). Maps A and B show households with at least one LLIN (purple) and no LLINs (orange). Maps C and D show the households that were reportedly sprayed (green) and not sprayed (orange). IRS, indoor residual spraying; LLIN, long-lasting insecticidal net; MDA, mass drug administration.
(TIF)

S1 Table Procedures conducted during the four MDA rounds and reactive focal MDAs implemented in Magude (2015–2018). MDA, mass drug administration.
(DOCX)

S2 Table Parasite prevalence and intervention coverage estimates reported during the annual cross-sectional surveys conducted at the end of the transmission season (May) in 2015–2018 in Magude district. Parasite prevalence and individual-level intervention coverage estimates were calculated as weighted proportions (if estimated for a subsample of the study population) or unweighted proportions (if estimated for the entire study population) for all age groups, and stratified by diagnostic method or age groups where applicable.
(DOCX)

S3 Table Sociodemographic information of Magude’s population collected during the baseline population census (2015) and during MDA rounds 1, 2, 3, and 4 for the individuals treated (Treat.), excluded (Excl.), and missing (Mis.). Information on the individuals who were missed during the MDA was obtained from the census closest to the MDA for those who were censed (32.2% in MDA1, 29.6% in MDA2, 24.5% in MDA3, 23.2% in MDA4). MDA, mass drug administration.
(DOCX)

S4 Table ITS coefficients to evaluate the impact of the interventions deployed during phase I (August of 2015–August 2017) and phase II (September 2017–June 2018) of the Magude project on the weekly number of malaria cases aggregated at district level. Level coefficients estimate the level change in the expected number of weekly malaria cases in the period immediately following the implementation of phase I (August 2015) and phase II interventions (September 2017). Trend coefficients represent the change in the trend of the expected number of malaria cases per week, relative to the trend in the previous period. ITS, interrupted time series.
(DOCX)

S5 Table AEs reported through the passive pharmacovigilance system of the MoH during the MDA rounds in Magude district. AE, adverse event; MDA, mass drug administration; MoH, Ministry of Health.
(DOCX)

S1 Appendix Study questionnaire used during the MDA rounds in Magude in Portuguese (original version), and translated into English. MDA, mass drug administration.
(DOCX)

S2 Appendix Study questionnaire used during the malaria cross-sectional surveys conducted in Magude in Portuguese (original version), and translated into English. (DOC)

S3 Appendix ITS model structure and description. ITS, interrupted time series.
(DOCX)

## Abbreviations

AE: adverse event
ANC: antenatal clinic
CHIRPS: Climate Hazards Group InfraRed Precipitation with Station data
CHW: community health worker
CI: confidence interval
CISM: Centro de Investigação em Saúde de Manhiça (Manhiça Health Research Center)
DDT: dichlorodiphenyltrichloroethane
DHAp: dihydroartemisinin-piperaquine
DHIS2: District Health Information System 2
EVI: enhanced vegetation index
FDC: Fundação para o Desenvolvimento da Comunidade
GBM: Goodbye Malaria
GEE: generalized estimating equations
GMPD: geometric mean parasite density
GTS: Global Technical Strategy
HF: health facility
HFCA: health-facility catchment area
HRP2: histidine-rich protein 2
IPTp: intermittent preventive treatment for pregnant women
IRS: indoor residual spraying
ITS: interrupted time series
LLIN: long-lasting insecticidal net
LSDI: Lubombo Spatial Development Initiative
MDA: mass drug administration
MODIS: moderate resolution imaging spectroradiometer, MoH, Ministry of Health
NMCP: National Malaria Control Program
NOAA: National Oceanic and Atmospheric Administration
PAR: population at risk
QIC: quasi-likelihood under the independence model criterion
RDT: rapid diagnostic test
RRS: rapid reporting system
SAE: serious adverse event
rfMDA: reactive focal mass drug administration
TPR: test positivity rate
WHO: World Health Organization
