# Supplementary figures and images for: A multiphase program for malaria elimination in southern Mozambique (the Magude project): A before-after study
Source: PLoS Med. 2020 Aug 14;17(8):e1003227. doi: 10.1371/journal.pmed.1003227 (PMC7428052; doi:10.1371/journal.pmed.1003227)

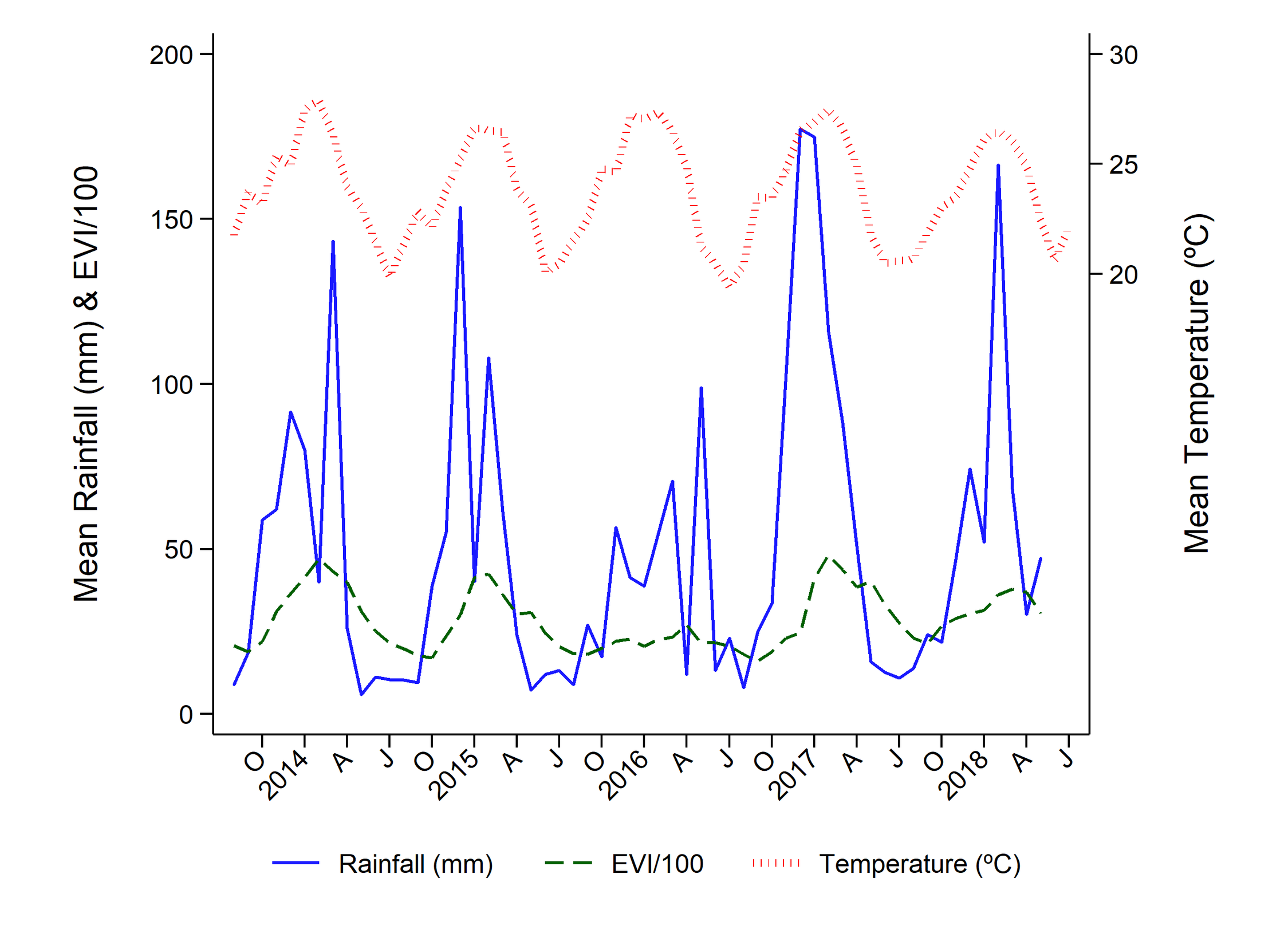

Supplement: S1 Fig — Rainfall raster data were obtained from the CHIRPS. EVI (MOD13A3) raster files were retrieved from MODIS satellite data. Data from every raster file per month were extracted for every household in Magude. Daily average temperature estimates were obtained from the NOAA collected by the Maputo Weather Station (station ID 673410) and aggregated monthly. CHIRPS, Climate Hazards Group InfraRed Precipitation with Station data; EVI, enhanced vegetation index; MODIS, moderate resolution imaging spectroradiometer; NOAA, National Oceanic and Atmospheric Administration. (TIF) [file pmed.1003227.s002.tif]

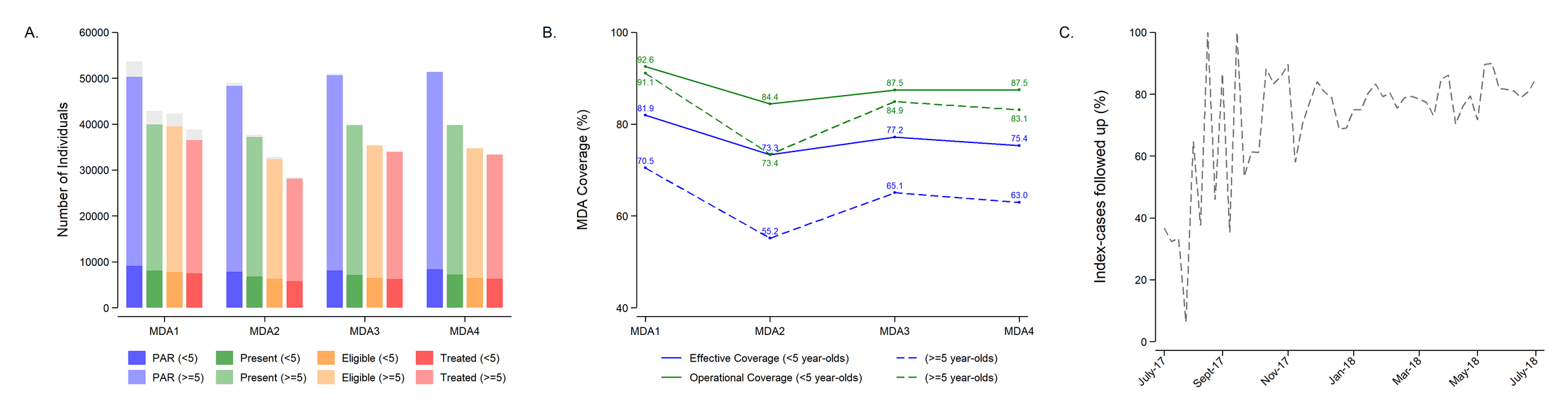

Supplement: S2 Fig — (A) Age-stratified coverage cascade of the population groups considered for the estimation of the effective and operational coverage of each MDA round (blue, PAR; green, present at the time of the MDA visit; orange, eligible for DHAp treatment; red, treated with DHAp; gray, missing information for age in any category). (B) Effective and operational coverage for <5 and ≥5-year-olds per MDA round. (C) Percentage of index cases detected at the HF/CHW for which an rfMDA response at the index case household was conducted. CHW, community health worker; DHAp, dihydroartemisinin-piperaquine; rfMDA, reactive focal mass drug administration; MDA, mass drug administration; PAR, population at risk; rfMDA, reactive focal mass drug administration. (TIF) [file pmed.1003227.s003.tif]

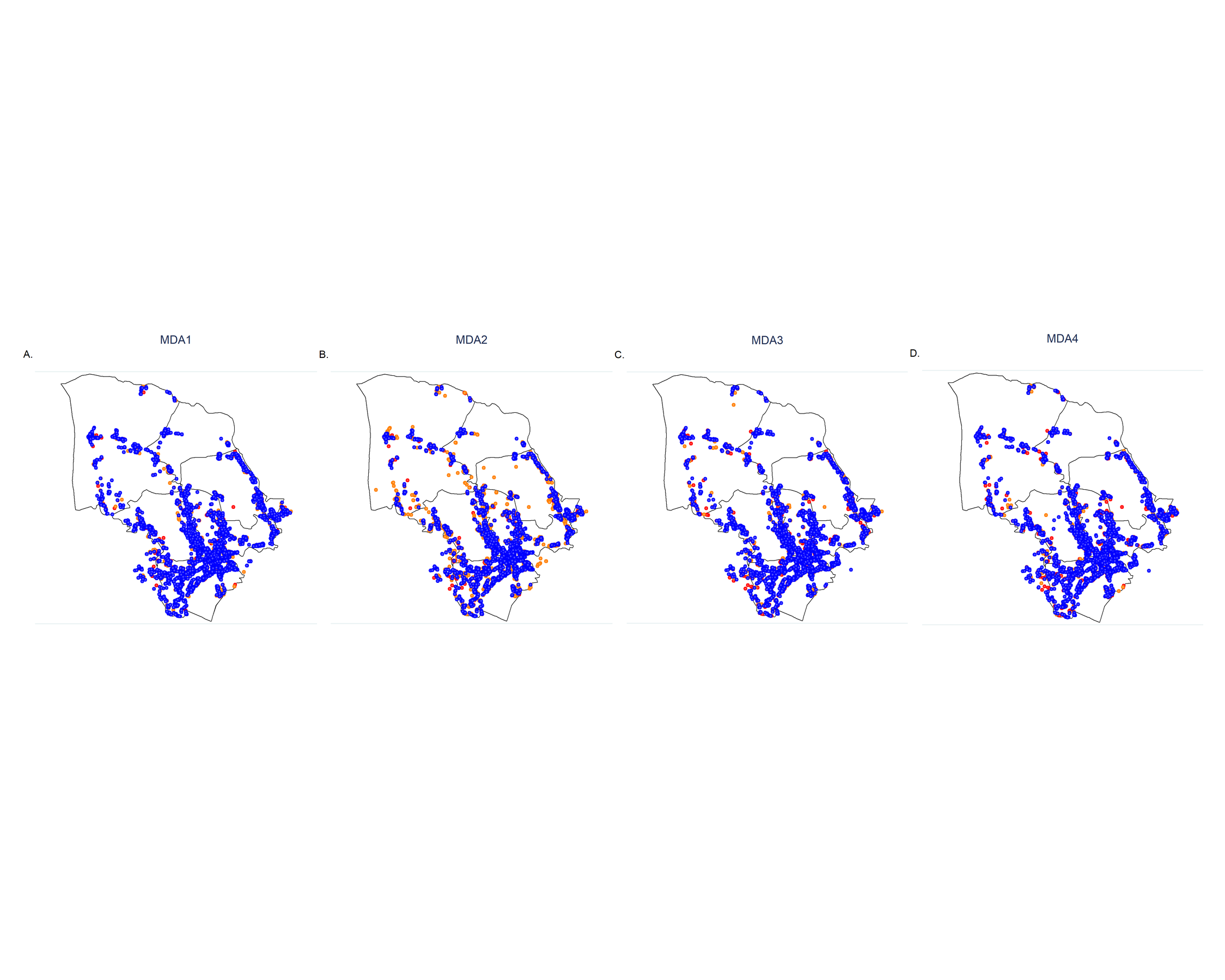

Supplement: S3 Fig — Maps of households covered by MDA1 (A), MDA2 (B), MDA3 (C), and MDA4 (D). Each map presents the households identified during the census of 2015 and 2016 that were not visited by the MDAs (red), households visited by the MDAs where no members were treated (orange), and households where at least one member was treated (blue). MDA, mass drug administration. (TIF) [file pmed.1003227.s004.tif]

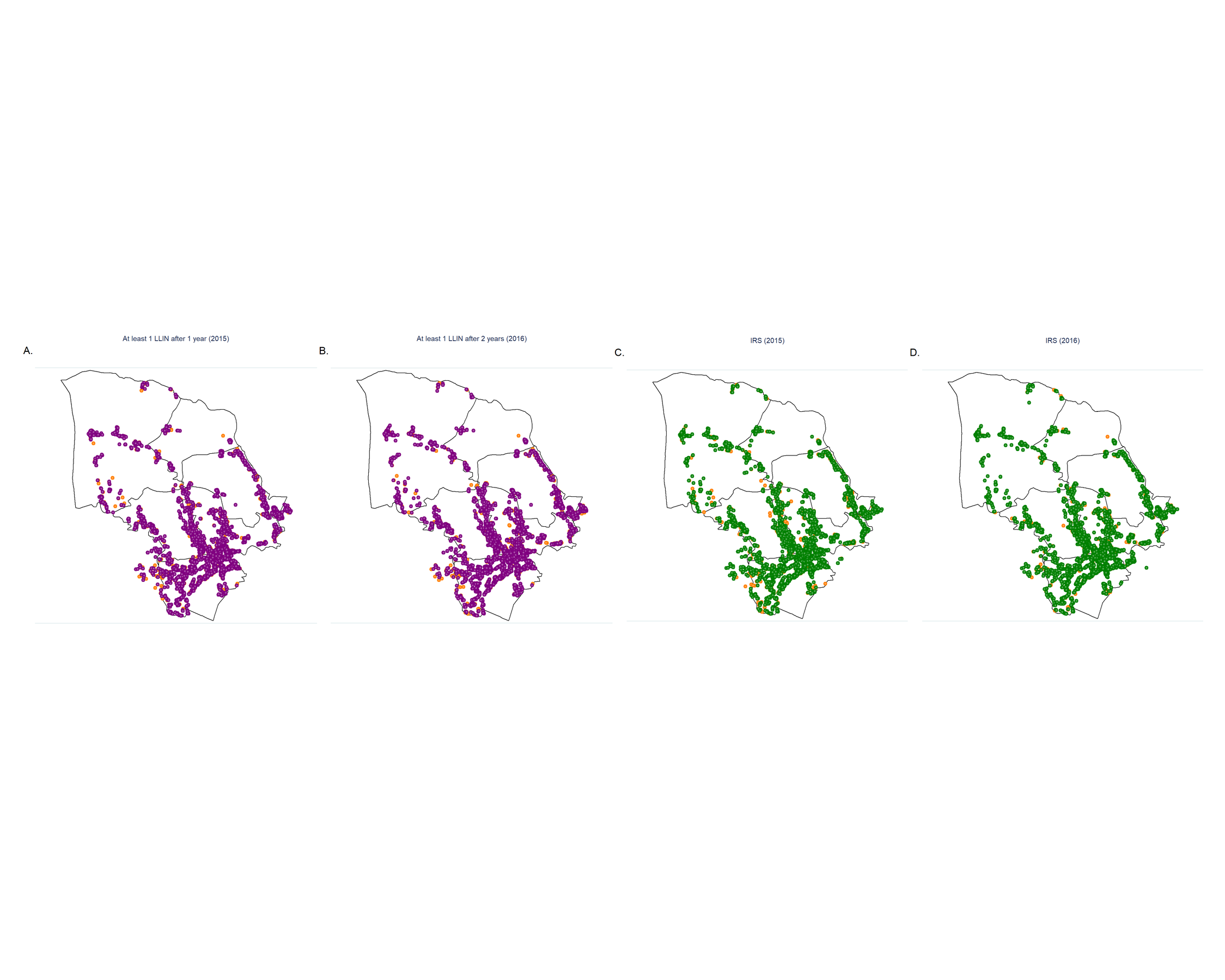

Supplement: S4 Fig — Maps of households that reported owning at least one LLIN one and two years after the LLIN distribution that took place in May of 2014 (A) and (B), and households that reported receiving IRS in the previous 12 months (C) and (D). Information on LLIN ownership was obtained from all the households censed during the census conducted in 2015 and 2016. Information on IRS was obtained from all households that participated in the MDA campaigns that took place immediately after the IRS campaign (i.e., MDA1 to evaluate the coverage of IRS in 2015, and MDA3 for the IRS of 2016). Maps A and B show households with at least one LLIN (purple) and no LLINs (orange). Maps C and D show the households that were reportedly sprayed (green) and not sprayed (orange). IRS, indoor residual spraying; LLIN, long-lasting insecticidal net; MDA, mass drug administration. (TIF) [file pmed.1003227.s005.tif]
