# Supplementary material for: A multiphase program for malaria elimination in southern Mozambique (the Magude project): A before-after study
Source: PLoS Med. 2020 Aug 14;17(8):e1003227. doi: 10.1371/journal.pmed.1003227 (PMC7428052; doi:10.1371/journal.pmed.1003227)
Supplement: S1 Table — MDA, mass drug administration. (DOCX) [file pmed.1003227.s006.docx]

**S.1. Table: Procedures conducted during the four Mass Drug Administration (MDA) rounds and reactive focal MDAs implemented in Magude (2015-18)**

|  | MDA-1  (Nov 2015) | MDA-2  (Jan-Feb 2016) | MDA-3  (Dec 2016) | MDA-4  (Jan-Feb 2017) | rfMDA  (July 2017- June 2018) |
| --- | --- | --- | --- | --- | --- |
| **Informed Consent** | Written consent obtained from all participants or their guardians (for participants <18 years old) and written assent for 12-17 years old. | | | | |
| **Inclusion criteria** | >6 months-old, >5kg, not pregnant in 1^st^ trimester, not severely ill | | | | |
| **Per-Protocol**  **Exclusion Criteria** | ≤ 6months-old, ≤5kg, first trimester pregnancies, severely ill | | | | |
| **Additional**  **Exclusion Criteria** |  | >65-Year-olds  All trimesters pregnant women | Pregnant women on IPTp  Contraindicated medications (as defined in the DHAp brochure) | |  |
| **Standardized Questionnaire** | For all households and individuals (full information for individuals that were present and basic information for those that were absent) | | | | |
| **Directly Observed Treatment** | Day 1 only | | | | |
| **Medication Regimen** | - 6 months - <2 y (5-12kg): 1 tablet per day (160mg/20mg) for 3 days - 2 -< 8 y (13-23kg): 1 tablet per day (320mg/40mg) for 3 days - 8 -<12 y (24-35kg): 2 tablets per day (320mg/40mg) for 3 days - ≥12 y (36-75kg): 3 tablets per day (320mg/40mg) for 3 days - ≥12 y (>75kg): 4 tablets per day (320mg/40mg) for 3 days | | | | |
| **Malaria Diagnostic Methods used** | RDT (all consenting participants) | RDT (random subsample) | Not performed | Not performed | RDT (index-case contacts) |
| **Pregnancy Testing Procedures** | All consenting women of reproductive age (12-49 years-old). | | All consenting women of reproductive age (12-49 year-olds), and increased efforts to increase pregnancy test acceptance | | |
| **Adherence Assessment on Day 4** | Random sample of MDA1 participants (N=511) | Random sample of MDA2 participants (N=1,134) | Not performed | Not performed | Not performed |
