# Supplementary material for: A multiphase program for malaria elimination in southern Mozambique (the Magude project): A before-after study
Source: PLoS Med. 2020 Aug 14;17(8):e1003227. doi: 10.1371/journal.pmed.1003227 (PMC7428052; doi:10.1371/journal.pmed.1003227)
Supplement: S2 Table — Parasite prevalence and individual-level intervention coverage estimates were calculated as weighted proportions (if estimated for a subsample of the study population) or unweighted proportions (if estimated for the entire study population) for all age groups, and stratified by diagnostic method or age groups where applicable. (DOCX) [file pmed.1003227.s007.docx]

**S2 Table: Parasite prevalence and intervention coverage estimates reported during the annual cross-sectional surveys conducted at the end of the transmission season (May) in 2015-2018 in Magude district**. Parasite prevalence and individual-level intervention coverage estimates were calculated as weighted proportions (if estimated for a sub-sample of the study population) or unweighted proportions (if estimated for the entire study population) for all age groups, and stratified by diagnostic method or age groups where applicable.

|  | **May 2015** | | **May 2016** | | **May 2017** | | **May 2018** | |
| --- | --- | --- | --- | --- | --- | --- | --- | --- |
| Number of participants | **1035** | | **1657** | | **3865** | | **3354** | |
|  | **n / N** | **% (95% CI)** | **n / N** | **% (95% CI)** | **n / N** | **% (95% CI)** | **n / N** | **% (95% CI)** |
| Infection prevalence^¶^ (N=Number of participants tested for each specific diagnostic method) | | | | | | | | |
| Microscopy | 68/992 | 7.1 (5.2, 9.6) | 14/1642 | 0.9 (0.5, 1.6) | 32/3445 | 0.8 (0.5, 1.2) | 32/3195 | 1.2 (0.7, 2.1) |
| <5 | 37/604 | 7.1 (5.1, 9.9) | 6/796 | 0.6 (0.1, 2.7) | 22/2353 | 1.1 (0.7, 1.8) | 11/1452 | 0.7 (0.4, 1.3) |
| ≥5 | 31/388 | 7.1 (4.9, 10.2) | 8/842 | 1.0 (0.5, 1.9) | 10/1077 | 0.6 (0.3, 1.3) | 21/1743 | - 1. (0.6, 2.1) |
| RDT | 91/1010 | 9.1 (7.0, 11.8) | 22/1651 | 1.5 (1.0, 2.5) | 109/3848 | 2.6 (2.0, 3.4) | 51/3327 | 1.4 (0.9, 2.2) |
| <5 | 50/616 | 9.8 (7.4, 13.0) | 10/807 | 1.9 (0.8, 4.5) | 71/2625 | 3.1 (2.3, 4.1) | 22/1509 | 1.4 (0.9, 2.1) |
| ≥5 | 41/394 | 9.0 (6.6, 12.3) | 12/840 | 1.4 (0.8, 2.5) | 38/1207 | 2.4 (1.7, 3.5) | 29/1818 | 1.4 (0.8, 2.43) |
| Afebrile infections± (N=Number of infections for which there was no missing clinical information) | | | | | | | | |
| Microscopy | 45/65 | 69.2 (56.6, 80.1) | 10/14 | 71.4 (41.9, 91.6) | 18/32 | 56.3 (37.7, 73.6) | 11/20 | 55.0 (31.5, 76.9) |
| <5 | 23/36 | 63.9 (46.2, 79.2) | 4/6 | 66.7 (22.3, 95.7) | 12/22 | 54.6 (32.2, 75.6) | 3/6 | 50 (11.8, 88.2) |
| ≥5 | 22/29 | 75.9 (56.5, 89.7) | 6/8 | 75 (34.9, 96.8) | 6/10 | 60 (26.2, 87.8) | 8/14 | 57.1 (28.9, 82.3) |
| RDT | 62/87 | 71.3 (60.6, 80.5) | 15/22 | 68.2 (45.1, 86.1) | 69/109 | 63.3 (53.5, 72.3) | 22/31 | 71.0 (52.0, 85.8) |
| <5 | 31/49 | 63.3 (48.3, 76.6) | 5/10 | 50 (18.7, 81.3) | 43/71 | 60.6 (48.3, 72) | 9/13 | 69.2 (38.6, 90.9) |
| ≥5 | 31/38 | 81.6 (65.7, 92.3) | 10/12 | 83.3 (51.6, 97.9) | 26/38 | 68.4 (51.4, 82.5) | 13/18 | 72.2 (46.5, 90.3) |
| Geometric Mean Parasite densities (N=Number of Infections, GMPDs presented in % column) | | | | | | | | |
| Microscopy | 68 | 931 (452, 1917) | 14 | 969 (255, 3683) | 32 | 1078 (484, 2401) | 32 | 1090 (471, 2523) |
| <5 | 37 | 2002 (766, 5235) | 5 | 236 (8, 7034) | 14 | 998 (278, 3578) | 7 | 1756 (390, 7908) |
| ≥5 | 31 | 374 (130, 1075) | 9 | 2127 (562, 8053) | 18 | 1145 (366, 3577) | 25 | 953 (340, 2677) |
| Interventions (N=Number of individuals without missing information for each variable) | | | | | | | | |
| Slept under a bed net the previous night^¶^ | 433/1035 | 40.9 (36.7, 45.3) | 1066/1590 | 64.4 (61.6, 67.2) | 2872/3832 | 72.2 (70.1, 74.3) | 2441/3351 | 70.6 (68.0, 73.1) |
| <5 | 265/625 | 39.5 (35.4, 43.8) | 501/705 | 68.6 (63.9, 73.0) | 1617/2049 | 80.8 (79.0, 82.5) | 983/1279 | 77.3 (74.8, 79.5) |
| ≥5 | 168/410 | 41.2 (36.3, 46.4) | 562/881 | 63.7 (60.4, 66.8) | 1245/1767 | 70.6 (68.0, 73.0)* | 1458/2072 | 69.3 (66.3, 72.3)* |
| Reported fever in previous 30 days ^¶^ | 357/1011 | 29.9 (26.1, 34.2) | 235/1657 | 11.2 (9.6, 13.1) | 681/3863 | 13.2 (11.7, 14.8) | 648/3354 | 18.4 (16.3, 20.6) |
| <5 | 236/616 | 34.2 (30.2, 38.4) | 123/745 | 11.5 (8.9, 14.7) | 406/2061 | 17.2 (15.4, 19.2) | 286/1281 | 22.2 (20.0, 24.7) |
| ≥5 | 121/395 | 29.1 (24.5, 34.1) | 111/908 | 11.2 (9.3, 13.4) | 272/1786 | 12.4 (10.7, 14.3) | 362/2073 | 17.6 (15.2, 20.2) |
| Sought care for fever (of those reporting a fever in previous 30 days) ± | 226/327 | 69.1 (63.8, 74.1) | 122/209 | 58.4 (51.4, 65.1) | 349/563 | 62.0 (57.8, 66.0) | 481/628 | 76.6 (73.1, 79.9) |
| <5 | 165/218 | 75.7 (69.4, 81.2) | 70/105 | 66.7 (56.8, 75.6) | 223/348 | 64.1 (58.8, 69.1) | 224/280 | 80.0 (74.8, 84.5) |
| ≥5 | 61/109 | 56.0 (46.1, 65.5)* | 52/103 | 50.5 (40.5, 60.5) | 123/212 | 58.0 (51.1, 64.7) | 257/348 | 73.9 (68.9, 78.4) |

¶Weighted Proportion

±Unweighted Proportion

*Non-overlapping Confidence Intervals between <5 and >=5 years-old estimates for the same year
