# Supplementary material for: A multiphase program for malaria elimination in southern Mozambique (the Magude project): A before-after study
Source: PLoS Med. 2020 Aug 14;17(8):e1003227. doi: 10.1371/journal.pmed.1003227 (PMC7428052; doi:10.1371/journal.pmed.1003227)
Supplement: S3 Table — MDA, mass drug administration. (DOCX) [file pmed.1003227.s008.docx]

**S.3. Table:** Socio-demographic information of Magude’s population collected during the baseline population census (2015) and during MDA rounds 1, 2, 3 and 4 for the individuals treated (Treat.), excluded (Excl.) and missing (Mis.). Information on the individuals who were missed during the MDA was obtained from the census closest to the MDA for those who were censed (32.2% in MDA1, 29.6% in MDA2, 24.5% in MDA3, 23.2% in MDA4).

|  | **Census** | | **MDA1** | | | | | | **MDA2** | | | | | | **MDA3** | | | | | | **MDA4** | | | | | |
| --- | --- | --- | --- | --- | --- | --- | --- | --- | --- | --- | --- | --- | --- | --- | --- | --- | --- | --- | --- | --- | --- | --- | --- | --- | --- | --- |
|  | **N** | **%** | **Treat** | **%** | **Excl** | **%** | **Mis.** | **%** | **Treat** | **%** | **Excl** | **%** | **Mis.** | **%** | **Treat** | **%** | **Excl** | **%** | **Mis.** | **%** | **Treat** | **%** | **Excl** | **%** | **Mis.** | **%** |
| Age |  |  |  |  |  |  |  |  |  |  |  |  |  |  |  |  |  |  |  |  |  |  |  |  |  |  |
| <1 | 1724 | 3.6 | 1841 | 4.7 | 231 | 8.3 | 328 | 3.0 | 649 | 2.3 | 313 | 6.4 | 327 | 3.3 | 595 | 1.8 | 423 | 10.2 | 427 | 3.4 | 572 | 1.7 | 507 | 10.1 | 456 | 3.6 |
| 1-<5 | 6866 | 14.2 | 4256 | 11.0 | 142 | 5.1 | 1331 | 12.1 | 3937 | 13.9 | 126 | 2.6 | 1291 | 13.2 | 4276 | 12.6 | 16 | 0.4 | 1596 | 12.8 | 4323 | 13.0 | 202 | 4.0 | 1602 | 12.6 |
| 5-<15 | 14607 | 30.1 | 10136 | 26.1 | 269 | 9.7 | 3040 | 27.7 | 9301 | 32.8 | 99 | 2.0 | 2869 | 29.4 | 10833 | 31.9 | 223 | 5.4 | 3464 | 27.8 | 10640 | 31.9 | 221 | 4.4 | 3564 | 28.0 |
| 15-<40 | 16411 | 33.9 | 11001 | 28.4 | 1201 | 43.3 | 4473 | 40.8 | 8705 | 30.7 | 1337 | 27.3 | 3648 | 37.3 | 10349 | 30.5 | 1614 | 39.0 | 4643 | 37.3 | 10478 | 31.4 | 1925 | 38.5 | 4590 | 36.0 |
| >=40 | 8816 | 18.2 | 9258 | 23.9 | 708 | 25.5 | 1784 | 16.3 | 5483 | 19.3 | 2980 | 61.0 | 1628 | 16.7 | 7889 | 23.2 | 1865 | 45.0 | 2306 | 18.5 | 7343 | 22.0 | 2149 | 42.9 | 2523 | 19.8 |
| Unknown | 24 | 0.0 | 2306 | 5.9 | 225 | 8.1 | 7 | 0.1 | 316 | 1.1 | 34 | 0.7 | 5 | 0.1 | 1 | 0.0 | 1 | 0.0 | 8 | 0.1 | 0 | 0.0 | 1 | 0.0 | 5 | 0.0 |
| Gender |  |  |  |  |  |  |  |  |  |  |  |  |  |  |  |  |  |  |  |  |  |  |  |  |  |  |
| Male | 21775 | 44.9 | 16220 | 41.8 | 920 | 33.1 | 5433 | 49.6 | 12253 | 43.2 | 1160 | 23.7 | 4603 | 47.1 | 14744 | 43.4 | 1094 | 25.5 | 5771 | 46.4 | 14832 | 44.5 | 1139 | 22.8 | 5858 | 46.0 |
| Female | 26673 | 55.1 | 22562 | 58.2 | 1856 | 66.9 | 5530 | 50.4 | 16137 | 56.8 | 3729 | 76.3 | 5165 | 52.9 | 19199 | 56.6 | 3193 | 74.5 | 6673 | 53.6 | 18511 | 55.5 | 3862 | 77.2 | 6882 | 54.0 |
| Education |  |  |  |  |  |  |  |  |  |  |  |  |  |  |  |  |  |  |  |  |  |  |  |  |  |  |
| No formal education | 19012 | 49.9 | 10512 | 53.8 | 803 | 52.9 | 4165 | 46.2 | 7095 | 50.4 | 2394 | 73.1 | 3749 | 47.2 | 8094 | 54.0 | 1396 | 60.0 | 4957 | 49.3 | 7796 | 52.4 | 1600 | 59.3 | 5287 | 51.3 |
| 5th-9th | 13837 | 36.2 | 6577 | 33.7 | 547 | 36.1 | 3409 | 37.8 | 5203 | 36.9 | 666 | 20.3 | 2971 | 37.4 | 5074 | 33.9 | 691 | 29.7 | 3622 | 36.0 | 5182 | 34.8 | 798 | 29.6 | 3595 | 34.9 |
| 10-12th | 3420 | 9.0 | 1487 | 7.6 | 126 | 8.3 | 989 | 11.0 | 991 | 7.0 | 127 | 3.9 | 852 | 10.7 | 1029 | 6.9 | 169 | 7.3 | 1000 | 9.9 | 1102 | 7.4 | 200 | 7.4 | 962 | 9.3 |
| University or higher | 183 | 0.5 | 61 | 0.3 | 6 | 0.4 | 61 | 0.7 | 58 | 0.4 | 5 | 0.2 | 49 | 0.6 | 56 | 0.4 | 4 | 0.2 | 63 | 0.6 | 47 | 0.3 | 11 | 0.4 | 55 | 0.5 |
| Unknown | 1672 | 4.4 | 884 | 4.5 | 35 | 2.3 | 392 | 4.3 | 737 | 5.2 | 85 | 2.6 | 323 | 4.1 | 731 | 4.9 | 68 | 2.9 | 417 | 4.1 | 761 | 5.1 | 89 | 3.3 | 407 | 3.9 |
| Occupation |  |  |  |  |  |  |  |  |  |  |  |  |  |  |  |  |  |  |  |  |  |  |  |  |  |  |
| Farmer or fisherman | 5280 | 49.0 | 2928 | 57.2 | 298 | 58.5 | 1134 | 41.2 | 1796 | 53.6 | 1009 | 78.2 | 994 | 42.6 | 2277 | 59.5 | 539 | 63.2 | 1321 | 43.8 | 2109 | 56.3 | 712 | 68.0 | 1387 | 42.9 |
| Salesperson | 1075 | 10.0 | 413 | 8.1 | 49 | 9.6 | 313 | 11.4 | 287 | 8.6 | 82 | 6.4 | 241 | 10.3 | 263 | 6.9 | 89 | 10.4 | 325 | 10.8 | 280 | 7.5 | 106 | 10.1 | 424 | 13.1 |
| Construction | 1198 | 11.1 | 366 | 7.2 | 39 | 7.7 | 392 | 14.2 | 261 | 7.8 | 23 | 1.8 | 293 | 12.6 | 266 | 6.9 | 39 | 4.6 | 351 | 11.6 | 290 | 7.7 | 42 | 4.0 | 362 | 11.2 |
| Coal maker / lumberjack | 776 | 7.2 | 366 | 7.2 | 29 | 5.7 | 201 | 7.3 | 274 | 8.2 | 40 | 3.1 | 210 | 9.0 | 292 | 7.6 | 33 | 3.9 | 260 | 8.6 | 297 | 7.9 | 33 | 3.2 | 281 | 8.7 |
| Guard/police /military | 584 | 5.4 | 245 | 4.8 | 22 | 4.3 | 169 | 6.1 | 210 | 6.3 | 24 | 1.9 | 132 | 5.7 | 202 | 5.3 | 28 | 3.3 | 159 | 5.3 | 211 | 5.6 | 22 | 2.1 | 189 | 5.8 |
| Health Staff | 218 | 2.0 | 98 | 1.9 | 11 | 2.2 | 55 | 2.0 | 63 | 1.9 | 18 | 1.4 | 45 | 1.9 | 63 | 1.6 | 19 | 2.2 | 65 | 2.2 | 72 | 1.9 | 27 | 2.6 | 67 | 2.1 |
| Teacher | 443 | 4.1 | 219 | 4.3 | 17 | 3.3 | 118 | 4.3 | 140 | 4.2 | 16 | 1.2 | 133 | 5.7 | 129 | 3.4 | 29 | 3.4 | 166 | 5.5 | 154 | 4.1 | 25 | 2.4 | 141 | 4.4 |
| Miner | 115 | 1.1 | 13 | 0.3 | 4 | 0.8 | 52 | 1.9 | 15 | 0.4 | 4 | 0.3 | 27 | 1.2 | 19 | 0.5 | 4 | 0.5 | 38 | 1.3 | 20 | 0.5 | 4 | 0.4 | 36 | 1.1 |
| Other | 1086 | 10.1 | 467 | 9.1 | 40 | 7.9 | 319 | 11.6 | 305 | 9.1 | 74 | 5.7 | 257 | 11.0 | 318 | 8.3 | 73 | 8.6 | 330 | 10.9 | 315 | 8.4 | 76 | 7.3 | 344 | 10.6 |
| Number of residents |  |  |  |  |  |  |  |  |  |  |  |  |  |  |  |  |  |  |  |  |  |  |  |  |  |  |
| 1 | 1087 | 2.2 | 599 | 2.3 | 64 | 3.7 | 286 | 2.6 | 207 | 0.8 | 352 | 10.4 | 343 | 3.5 | 352 | 1.7 | 129 | 5.2 | 509 | 4.1 | 299 | 1.4 | 139 | 4.9 | 566 | 4.4 |
| 1-<5 | 10523 | 21.7 | 5234 | 20.1 | 454 | 26.0 | 2719 | 24.8 | 3487 | 13.4 | 1041 | 30.8 | 2808 | 28.7 | 3753 | 17.9 | 675 | 27.3 | 3734 | 30.0 | 3704 | 17.8 | 791 | 27.7 | 3851 | 30.2 |
| >=5 | 36838 | 76.0 | 20244 | 77.6 | 1225 | 70.3 | 7958 | 72.6 | 16229 | 62.2 | 1989 | 58.8 | 6617 | 67.7 | 16806 | 80.4 | 1672 | 67.5 | 8201 | 65.9 | 16777 | 80.7 | 1923 | 67.4 | 8323 | 65.3 |
