# Supplementary material for: A multiphase program for malaria elimination in southern Mozambique (the Magude project): A before-after study
Source: PLoS Med. 2020 Aug 14;17(8):e1003227. doi: 10.1371/journal.pmed.1003227 (PMC7428052; doi:10.1371/journal.pmed.1003227)
Supplement: S4 Table — Level coefficients estimate the level change in the expected number of weekly malaria cases in the period immediately following the implementation of phase I (August 2015) and phase II interventions (September 2017). Trend coefficients represent the change in the trend of the expected number of malaria cases per week, relative to the trend in the previous period. ITS, interrupted time series. (DOCX) [file pmed.1003227.s009.docx]

**S.4. Table: Interrupted time-series coefficients to evaluate the impact of the interventions deployed during phase I (August of 2015-August 2017) and phase II (September 2017-June 2018) of the Magude Project on the weekly number of malaria cases aggregated at district level**. Level coefficients estimate the level change in the expected number of weekly malaria cases in the period immediately following the implementation of phase I (August 2015) and phase II interventions (September 2017). Trend coefficients represent the change in the trend of the expected number of malaria cases per week, relative to the trend in the previous period.

|  | Exponentiated ß coefficients | 95% CI |
| --- | --- | --- |
| Intervention components: |  |  |
| Baseline Level (βo) | 200.04 | 186.83, 259.150* |
| Baseline Trend (β_1_) | 1.003 | 1.006, 0.046* |
| Phase 1^¶^ Level (β_2_) | 0.309 | 0.225, 0.425* |
| Phase 1 Trend (β_3_) | 0.996 | 0.991, 1.002 |
| Phase 2^±^ Level (β_4_) | 1.224 | 0.880, 1.703 |
| Phase 2 Trend (β_5_) | 0.994 | 0.981, 1.008 |
| Covariates |  |  |
| Mean rainfall (lag 1) | 1.001 | 0.999, 1.003 |
| Mean temperature (lag 1) | 0.988 | 0.959, 1.017 |
| Mean EVI (lag 1) | 1.445 | 1.293, 1.616* |
| Mean EVI (lag 2) | 0.964 | 0.878, 1.059 |
| Non-malaria cases | 1.0003 | 1.0002, 1.0005* |
| LLINs per capita (lag 1) | 0.692 | 0.425, 1.127 |

¶Phase I interventions: Two rounds of MDA with DHAp for two consecutive years + yearly IRS + variations in LLIN use as a result of community engagement

±Phase II interventions: rfMDA + yearly IRS + variations in LLIN use as a result of community engagement

*statistically significant at <0.05 level
