# Supplementary material for: A multiphase program for malaria elimination in southern Mozambique (the Magude project): A before-after study
Source: PLoS Med. 2020 Aug 14;17(8):e1003227. doi: 10.1371/journal.pmed.1003227 (PMC7428052; doi:10.1371/journal.pmed.1003227)
Supplement: S5 Table — AE, adverse event; MDA, mass drug administration; MoH, Ministry of Health. (DOCX) [file pmed.1003227.s010.docx]

**S.5. Table: Adverse events reported through the passive pharmacovigilance system of the Ministry of Health during the MDA rounds in Magude district.**

|  | MDA1 | | MDA2 | | MDA3 | | MDA4 | | All rounds | |
| --- | --- | --- | --- | --- | --- | --- | --- | --- | --- | --- |
|  | **N (col %)** | **% of treated (N=38792)** | **N (col %)** | **% of treated (N=28393)** | **N (col %)** | **% of treated (N=33943)** | **N (col %)** | **% of treated  (N=33356)** | **N (col %)** | **% of treated  (N=33356)** |
| Head ache | 31 (28) | 0.080 | 20 (27) | 0.070 | 15 (19) | 0.044 | 4 (24) | 0.012 | 70 (25) | 0.052 |
| Vomiting | 16 (15) | 0.041 | 12 (16) | 0.042 | 14 (18) | 0.041 | 4 (24) | 0.012 | 46 (17) | 0.034 |
| Fever | 13 (12) | 0.034 | 7 (9) | 0.025 | 6 (8) | 0.018 | 0 (0) | 0.000 | 26 (9) | 0.019 |
| Asthenia | 11 (10) | 0.028 | 6 (8) | 0.021 | 10 (13) | 0.029 | 3 (18) | 0.009 | 30 (11) | 0.022 |
| Abdominal pain | 6 (6) | 0.015 | 6 (8) | 0.021 | 6 (8) | 0.018 | 0 (0) | 0.000 | 18 (6) | 0.013 |
| Dizziness | 8 (7) | 0.021 | 3 (4) | 0.011 | 6 (8) | 0.018 | 1 (6) | 0.003 | 18 (6) | 0.013 |
| Anorexia | 2 (2) | 0.005 | 4 (5) | 0.014 | 5 (6) | 0.015 | 2 (12) | 0.006 | 13 (5) | 0.010 |
| Diarrhea | 3 (3) | 0.008 | 3 (4) | 0.011 | 1 (1) | 0.003 | 0 (0) | 0.000 | 7 (3) | 0.005 |
| Pruritus | 4 (4) | 0.010 | 2 (3) | 0.007 | 3 (4) | 0.009 | 0 (0) | 0.000 | 9 (3) | 0.007 |
| Arthralgia | 1 (1) | 0.003 | 4 (5) | 0.014 | 3 (4) | 0.009 | 1 (6) | 0.003 | 9 (3) | 0.007 |
| Nausea | 3 (3) | 0.008 | 2 (3) | 0.007 | (0) | 0.000 | (0) | 0.000 | 5 (2) | 0.004 |
| Tachycardia | 4 (4) | 0.010 | 1 (1) | 0.004 | 2 (3) | 0.006 | 0 (0) | 0.000 | 7 (3) | 0.005 |
| Dyspnea | 1 (1) | 0.003 | 2 (3) | 0.007 | 1 (1) | 0.003 | 0 (0) | 0.000 | 4 (1) | 0.003 |
| Chest pain | 2 (2) | 0.005 | 1 (1) | 0.004 | 1 (1) | 0.003 | 0 (0) | 0.000 | 4 (1) | 0.003 |
| Dysuria | 0 (0) | 0.000 | 1 (1) | 0.004 | 1 (1) | 0.003 | 0 (0) | 0.000 | 2 (1) | 0.001 |
| Blurred vision | 1 (1) | 0.003 | 0 (0) | 0.00 | 0 (0) | 0.000 | 1 (6) | 0.003 | 2 (1) | 0.001 |
| Coughing | 3 (3) | 0.008 | 0 (0) | 0.00 | (0) | 0.000 | (0) | 0.000 | 3 (1) | 0.002 |
| Somnolence | (0) | 0.00 | (0) | 0.00 | 1 (1) | 0.003 | 0 (0) | 0.000 | 1 (0) | 0.001 |
| Skin rash | (0) | 0.00 | (0) | 0.00 | 2 (3) | 0.006 | 1 (6) | 0.003 | 3 (1) | 0.002 |
| Total | 109 (100) |  | 74 (100) |  | 77 (100) |  | 17 (100) |  | 277 (100) |  |
