# Supplementary material for: A multiphase program for malaria elimination in southern Mozambique (the Magude project): A before-after study
Source: PLoS Med. 2020 Aug 14;17(8):e1003227. doi: 10.1371/journal.pmed.1003227 (PMC7428052; doi:10.1371/journal.pmed.1003227)
Supplement: S1 Appendix — MDA, mass drug administration. (DOCX) [file pmed.1003227.s011.docx]

**S1 Appendix:** Study questionnaire used during the Mass drug administration rounds in Magude in Portuguese (original version), and translated into English.

**Estudo: Administração Massiva de Tratamento Mensal de Dihidroartemisina-Piperaquina (DHA-PQP) com vista a eliminação da malária no distrito de Magude, Sul de Moçambique: Detecção Reactiva de Casos de Malária**

**Inquerito de estudo**

| **1. Identificação do agregado** | | | | | |
| --- | --- | --- | --- | --- | --- |
|  | Área | □Xinavane^1^ □Magude Sede □Panjane □Motaze □Mahele □Mapulanguene | | | |
|  | O agregado foi recenseado pela equipa do CISM? | | | Sim □ Não□ | |
|  | Agregado (e coordenadas GPS no formato electrónico) | | | \|__\|__\|__\|__\|-\|__\|__\|__\| | |
|  | Foi possivel efectuar a visita? | | | Sim □ Não□ | |
|  | Se não, especificar o motivo e passar para **Assinaturas** | | | □ Recusa □Ausência do chefe do agregado  □ Emigração dos residentes □ Outro (especifique) ________ | |
|  | Data da visita | | | \|__\|__\|/\|__\|__\|/\|__\|__\|__\|__\| | |
|  | Nome do chefe do agregado | | | \|__\|__\|__\|__\|__\|__\|__\|__\|__\|__\|__\|__\|__\|__\|__\|__\|__\|__\| | |
|  | Apelido do chefe do agregado | | | \|__\|__\|__\|__\|__\|__\|__\|__\|__\|__\|__\|__\|__\|__\|__\|__\|__\|__\| | |
|  | Perm ID do chefe do agregado | | | \|__\|__\|__\|__\|-\|__\|__\|__\|-\|__\|__\| | |
|  | Já alguma vez ouviu alguma mensagem de sensibilização sobre este campanha?  *Se não,***passar para a pergunta 12.** | | | Sim □ Não□ | |
|  | *Se sim*, através de que meio? | | □Rádio  □ Líderes comunitários  □ Reuniões com a comunidade  □No hospital ou unidade sanitária | | □Escola  □Igreja  □Microfone  □Panfletos/Poster  □ Outro ______________________ |
|  | Este agregado participou na ronda de AMT de Dezembro?^2^ | | | Sim □ Não□ | |
|  | Se não, porque não? | | □ Equipa não veio □ Não estavam em casa □ Recusaram □ Não sabe/outros | | |
|  | A casa foi fumigada nos últimos 12 meses? | | | Sim □ Não□ | |
|  | Se não, motivo? | | □ Equipa não veio □ Não estavam em casa □ Recusaram □ Não sabe/outros | | |

| **2. Identificação dos membros do agregado**  NOTA: Uma lista com as pessoas que foram censadas no agregado aparecerá no tablet. A informaçao terá de ser recolhida para cada membro da lista. Os novos membros, ou pessoas encontradas no momento da visita terão de ser adicionadas**.** | | | | | | | | | | | | | | | |
| --- | --- | --- | --- | --- | --- | --- | --- | --- | --- | --- | --- | --- | --- | --- | --- |
|  | Tipo de Visita | | | | | | | | Em casa □ Ponto fixo □ Açucareira /Plantações □  Mercado/Escola/Internato □ Outros □ | | | | | | |
|  | Nome do membro | | | | | | | | \|__\|__\|__\|__\|__\|__\|__\|__\|__\|__\|__\|__\|__\|__\|__\|__\|__\|__\|__\| | | | | | | |
|  | Apelido do membro | | | | | | | | \|__\|__\|__\|__\|__\|__\|__\|__\|__\|__\|__\|__\|__\|__\|__\|__\|__\|__\|__\| | | | | | | |
|  | Perm ID do membro | | | | | | | | \|__\|__\|__\|__\|-\|__\|__\|__\|-\|__\|__\| | | | | | | |
|  | A pessoa está presente no agregado? | | | | | | | | Sim □ Não□ | | | | | | |
|  | *Se não*, porque não?  **Passar as assinaturas** | | | | | | | | □ Ausente durante o dia (ex: trabalho, machamba, escola, etc)  □ Ausente por alguns dias (viagem)  □ Mudou de residência dentro de Magude  □ Emigrou há mais de um mês  □ Faleceu | | | | | | |
|  | A pessoa aceita participar no estudo? | | | | | | | | Sim □ Não□ | | | | | | |
|  | Se não, porque não?  **Passar as assinaturas** | | | | | | | | □ Não confia na equipe do estudo  □ Não gostou da experiencia passad  □ Outro | | | | | | |
|  | A pessoa é membro deste agregado? | | | | | | | | Sim □ Não□ | | | | | | |
|  | Relação com o chefe do agregado | | | | | \|__\| (1=Esposo/a; 2=Filho/a; 3=Irmão/a; 4=Pai/mãe; 5= Nora; 6= Cunhado/a; 7= Tio/a; 8= Neto; 9= Enteado; 10= Adoptado; 11= Primo; 12= Sobrinho; 13=Não família; 14=Outro) | | | | | | | | | |
|  | Data de nascimento | | | | | | | | | \|__\|__\|/\|__\|__\|/\|__\|__\|__\|__\| | | | | | |
|  | Idade | \|__\|__\| anos ou \|__\|__\| meses (para crianças menores de 1 ano) | | | | | | | | | | | | | |
|  | Gênero | | | | | | | | | | | Feminino □ Masculino □ | | | |
|  | **Mulheres em idade reprodutiva (12-49 anos)** | | | | | | | | | | | | | | |
|  | Está grávida? | | | | | | | | | | | Sim □ Não□ Não sabe □ | | | |
|  | - Se sim, há quanto tempo está grávida? | | | | | | | | | | | \|__\|__\| meses | | | |
|  | Se sim (cont.), tem ido as consultas pré-natais na Unidade Sanitária? | | | | | | | | | | | Sim □ Não□ | | | |
|  | Se sim (cont.), está a receber o Tratamento Intermitente Preventivo (TIP) de malária?^3^ | | | | | | | | | | | Sim □ Não□ | | | |
|  | Se sim toma TIP, quando foi a ultima dose? ^3^ | | | | | | | | | | | \|__\|__\|/\|__\|__\|/\|__\|__\|__\|__\| | | | |
|  | - Se não ou não sabe, fez o teste rápido de gravidez? | | | | | | | | | | | Sim □ Não□ | | | |
|  | Se não, por quê? | | | | | | | | | | | Recusa □ Não quero saber □ | | | |
|  | Se sim, resultado do teste rápido de gravidez  **NOTA**: Se for POSITIVO, NÃO MEDICAR | | | | | | | | | | | Positivo □ Negativo □ Inválido □ | | | |
|  | Teve febre nas últimas 24 horas? | | | | | | | | | | | Sim □ Não□ | | | |
|  | Tomou antimaláricos nos últimos 30 dias? | | | | | | | | | | | Sim □ Não□ | | | |
|  | Toma antirretrovirais? | | | | | | | | | | | Sim □ Não□ Não quer responder □ | | | |
|  | Em termos gerais, sente-se saudável? | | | | | | | | | | | Sim □ Não□ | | | |
|  | Dormiu sob uma rede mosquiteira na noite anterior? | | | | | | | | | | | Sim □ Não□ | | | |
|  | Se não, porquê? | | | □ Não tenho □ Não gosto □ Não está pendurada □ Faz calor  □ Outro _______________________________ | | | | | | | | | | | |
| **Diagnóstico de malária ^4^** | | | | | | | | | | | | | | | |
|  | Foi feito o teste rápido de malária? **^4^** | | | | | | | | | | | Sim □ Não□ | | | |
|  | Se sim, resultado do teste rápido de malária **^4^** | | | | | | | | | | | Positivo □ Negativo □ | | | |
|  | Foi colhido um papel de filtro? **^4^** | | | | | | | | | | | Sim □ Não□ | | | |
|  | Se sim, colar o NIDA **^4^**  (ou ler o codigo de barras usando a camara do tablet) | | | | | | | | | | |  | | | |
| **Administração do Medicamento de estudo** | | | | | | | | | | | | | | | |
|  | A pessoa cumpre os critérios de inclusão para tomar o medicamento? | | | | | | | | | Sim □ Não□ | | | | | |
|  | Se não, assinalar o critério de exclusão. | | | | | | □ Criança menor de 6 meses  □ Mulher grávida no primeiro trimestre  □ Mulher grávida em TIP  □ Está em medicação contraindicada  □Tem doença grave e foi referido a Unidade Sanitária | | | | | | | | |
|  | Tomou a primeira dose de DHA-PQP na presença do Inquiridor? | | | | | | | | | | | | | Sim □ Não□ | |
|  | Se sim, dosagem administrada? | | | | □5kg – 12kg  (6m-2 anos)  (rosa) | | | □13kg – 23kg  (2-8 anos)  (verde) | | | □24kg – 35kg  (8-12 anos)  (laranja) | | □36kg – 74kg  (mais de 12 anos)  (violeta) | | □>75kg  (preta) |
|  | Se sim, numero de comprimidos administrados (toma do primeiro dia) | | | | | | | | | | | | | \|__\| | |
|  | Se não, especificar a razão da recusa do participante? | | □ Não está doente  □ Tem medo do medicamento ou dos seus possíveis efeitos □ Não acredita que o medicamento previna malária  □ Ouviu rumores negativos acerca do medicamento  □ Crenças religiosa/culturais  □ Não confia na equipa de estudo  □ Está sob os efeitos do álcool  □ Ainda não comeu  □ Outro (specificar) _________________________________________ | | | | | | | | | | | | |
|  | O participante vomitou? | | | | | | Sim □ Não□ | | | | | | | | |
|  | Se o participante vomitou, repetiu o tratamento? | | | | | | Sim □ Não□ | | | | | | | | |
|  | Se não, porque não? | | | | | | Recusa □ Receio de vomitar novamente □ Outro: _____________ | | | | | | | | |
| **Assinaturas** | | | | | | | | | | | | | | | |
|  | Código e Assinatura Inquiridor | | | | | | ___________________________________ \|__\|__\|__\| | | | | | | | | |
|  | Código e Assinatura Pessoal de Laboratório | | | | | | ___________________________________ \|__\|__\|__\| | | | | | | | | |

**Controle de versões**

1. Perguntado nas rondas de AMT3 e AMT4
2. Perguntado nas rondas AMT2 e AMT4
3. Perguntado nas rondas AMT2, AMT3 e AMT4
4. Perguntado nas rondas de AMT1 e 2

**Study: Monthly mass drug administration with dihydroartemisinin-piperaquine (DHA-PQP) to eliminate malaria in the district of Magude, southern Mozambique**

**Study questionnaire**

(English transation)

| **1. Household identification** | | | | | | | | | |
| --- | --- | --- | --- | --- | --- | --- | --- | --- | --- |
|  | Area | □Xinavane^1^ □Magude Sede □Panjane □Motaze □Mahele □Mapulanguene | | | | | | | |
|  | Was the household censed by CISM? | | | | | | Yes □ No □ | | |
|  | Household number (and GPS coordinates on electronic questionnaire) | | | | | | \|__\|__\|__\|__\|-\|__\|__\|__\| | | |
|  | Was it possible to visit the household? | | | | | | Yes □ No □ | | |
|  | If no, specify the reason and sign the questionnaire | | | | □ Refused □ Absenteeism of head of household  □ Emigration of the residents □ Other (specify) __________________ | | | | |
|  | Date of visit | | | | | | \|__\|__\|/\|__\|__\|/\|__\|__\|__\|__\| | | |
|  | Name of head of household | | | | | | \|__\|__\|__\|__\|__\|__\|__\|__\|__\|__\|__\|__\|__\|__\|__\|__\|__\| | | |
|  | Surname of head of household | | | | | | \|__\|__\|__\|__\|__\|__\|__\|__\|__\|__\|__\|__\|__\|__\|__\|__\|__\| | | |
|  | Permanent identification number of the head of household | | | | | | \|__\|__\|__\|__\|-\|__\|__\|__\|-\|__\|__\| | | |
|  | Has the head of household heard any community engagement messages on the MDA campaign? *If no ,***skip to question 12.** | | | | | | Yes □ No □ | | |
|  | *If yes,* through what means? | | | □Radio  □ Community leaders  □ Community meetings  □At the hospital or health facility | | | | □School  □Church  □Microphone  □Pamphlets / Posters  □ Other (specify) ___________________ | |
|  | Did this household participate in the MDA rounds that took place in December? ^2^ | | | | | | | | Yes □ No □ |
|  | *If no*, why not? | | □ The MDA team did not come □ They were not home □ Refused □Does not know /other | | | | | | |
|  | Has the household been sprayed in the preceding 12 months? | | | | | Yes □ No □ | | | |
|  | If no, why not? | | □ The spraying team did not come □ They were not home □ Refused □ Does not know /other | | | | | | |

| **2. Identification of the household members**  Note: A list of all members censed for the specific household will appear. Information should be collected for all members, and any other person found or reported to also live in the household who is not in the list should be added. | | | | | | | | | | | | | | | | |
| --- | --- | --- | --- | --- | --- | --- | --- | --- | --- | --- | --- | --- | --- | --- | --- | --- |
|  | Type of visit | | | At home □ Fixed point^1^ □ Sugar plantation □ Market/Boarding school □ Other □ | | | | | | | | | | | | |
|  | Name of participant | | | | | | | | | \|__\|__\|__\|__\|__\|__\|__\|__\|__\|__\|__\|__\|__\|__\|__\|__\|__\|__\|__\| | | | | | | |
|  | Surname of participant | | | | | | | | | \|__\|__\|__\|__\|__\|__\|__\|__\|__\|__\|__\|__\|__\|__\|__\|__\|__\|__\|__\| | | | | | | |
|  | Permanent identification number of participant | | | | | | | | | \|__\|__\|__\|__\|-\|__\|__\|__\|-\|__\|__\| | | | | | | |
|  | Is the person present in the household? | | | | | | | | | Yes □ No □ | | | | | | |
|  | *If no*, why not?  **Skip to the signatures section** | | | | | | | | | □ Absent during the day (ex: at work, plantation, school, etc)  □Absent for some days (on a trip)  □ Changed to another residency in Magude  □ Emigrated more than 1 months ago  □ Died | | | | | | |
|  | Did the person accept to participate in this study? | | | | | | | | | Yes □ No □ | | | | | | |
|  | *If no,* why not?  **Skip to the signatures section** | | | | | | | | | □ Does not trust the study team  □ Did not like the previous MDA experiences  □ Other | | | | | | |
|  | Is the person part of this household? | | | | | | | | | Yes □ No □ | | | | | | |
|  | State the relationship with the head of the household | | | | | | \|__\| (1=Partner; 2=Child; 3=Sibling; 4=Parent; 5= daughter in law; 6= Sibling in law; 7=Uncle/aunt ; 8= Grandchild; 9= Step-child; 10= Adopted; 11= Cousin; 12= Nephew/niece; 13=Not related; 14=Other) | | | | | | | | | |
|  | Date of birth | | | | | | | | | | \|__\|__\|/\|__\|__\|/\|__\|__\|__\|__\| | | | | | |
|  | Age | \|__\|__\| in years or \|__\|__\| in months (if the child is <1-year-old) | | | | | | | | | | | | | | |
|  | Sex | | | | | | | | | | | | Female □ Male □ | | | |
|  | **Women of reproductive age (12-49 years-old)** | | | | | | | | | | | | | | | |
|  | Is she pregnant? | | | | | | | | | | | | Yes □ No □ Does not know □ | | | |
|  | - *If yes*, for how long has she been pregnant? | | | | | | | | | | | | \|__\|__\| months | | | |
|  | *If yes* (cont.), has she gone to the antenatal clinic already? | | | | | | | | | | | | Yes □ No □ | | | |
|  | *If yes (*cont.), is she taking IPTp?^3^ | | | | | | | | | | | | Yes □ No □ | | | |
|  | *If she has taken IPTp*, when was the last dose taken? ^3^ | | | | | | | | | | | | \|__\|__\|/\|__\|__\|/\|__\|__\|__\|__\| | | | |
|  | - If she is not pregnant or does not know, did she do a pregnancy test? | | | | | | | | | | | | Yes □ No □ | | | |
|  | *If no*, why not? | | | | | | | | | | | | Refuses □ Does not want to know □ | | | |
|  | *If yes*, specify the result of the test  **NOTE**: If the result is POSITIVE, DO NOT MEDICATE | | | | | | | | | | | | Positive □ Negative □ Invalid □ | | | |
|  | Did the participant have fever in the preceding 24 hours? | | | | | | | | | | | | Yes □ No □ | | | |
|  | Did the participant take antimalarial drugs in the preceding 30 days? | | | | | | | | | | | | Yes □ No □ | | | |
|  | Is the participant under antiretroviral treatment? | | | | | | | | | | | | Yes □ No□ Prefers not to answer □ | | | |
|  | In general terms, does the participant feel healthy? | | | | | | | | | | | | Yes □ No □ | | | |
|  | Did the participant sleep under a bed net the night before the visit? | | | | | | | | | | | | Yes □ No □ | | | |
|  | *If no*, why not? | | | | □ Does not have one □ Does not like it □ Is not hanging □ It’s hot  □ Other (specify) ____________________________ | | | | | | | | | | | |
| **Malaria diagnostic ^4^** | | | | | | | | | | | | | | | | |
|  | Was a malaria rapid diagnostic test (RDT) conducted? **^4^** | | | | | | | | | | | | Yes □ No □ | | | |
|  | *If yes*, specify the result of the RDT **^4^** | | | | | | | | | | | | Positive □ Negative □ | | | |
|  | Were filter paper samples collected? **^4^** | | | | | | | | | | | | Yes □ No □ | | | |
|  | *If yes*, stick the sample ID barcode **^4^**  (or read the barcode using the tablet’s camera) | | | | | | | | | | | |  | | | |
| **Drug administration** | | | | | | | | | | | | | | | | |
|  | Is the participant eligible to take DHA-PQP? | | | | | | | | | | Yes □ No□ | | | | | |
|  | *If no*, select the exclusion criteria. | | | | | | | □ Child younger than 6-months of age  □ Pregnant woman in the first trimester  □ Pregnant woman on IPTp^3^  □ Taking contraindicated medication  □ Is severely ill and was referred to the nearest health facility | | | | | | | | |
|  | Did the participant take the drug in front of the field worker? | | | | | | | | | | | | | | Yes □ No □ | |
|  | *If yes*, what was the dose administered? | | | | | □5kg – 12kg  (6m-2 years)  (pink) | | | □13kg – 23kg  (2-8 years)  (green) | | | □24kg – 35kg  (8-12 years)  (orange) | | □36kg – 74kg  (> 12 years)  (purple) | | □>75kg  (black) |
|  | *If yes*, specify the number of pills administered (on day 1) | | | | | | | | | | | | | | \|__\| | |
|  | *If no,* specify the reason for refusal provided by the participant? | | □ S/he is not ill  □ S/he is scared of the drug or its adverse events □ Does not believe that the drug can prevent against malaria  □ Heard negative rumors against the drug  □ Religious / cultural beliefs  □ Does not trust the study team  □ Drunkenness  □ Has not yet eaten  □ Other (specify) _________________________________________ | | | | | | | | | | | | | |
|  | Did the participant vomit? | | | | | | | Yes □ No □ | | | | | | | | |
|  | If yes, was the treatment repeated? | | | | | | | Yes □ No □ | | | | | | | | |
|  | If no, why not? | | | | | | | Refuses □ Does not want to vomit again □ Other (specify)__________ | | | | | | | | |
| **Signatures** | | | | | | | | | | | | | | | | |
|  | Code and signature of the data collector | | | | | | | ___________________________________ \|__\|__\|__\| | | | | | | | | |
|  | Code and signature of the laboratory technician | | | | | | | ___________________________________ \|__\|__\|__\| | | | | | | | | |

**Version control**

1. Asked during MDAs3 and 3
2. Asked during MDAs2 and 4
3. Asked during MDAs 2, 3 and 4
4. Asked during MDAs 1 and 2
