# Supplementary material for: A multiphase program for malaria elimination in southern Mozambique (the Magude project): A before-after study
Source: PLoS Med. 2020 Aug 14;17(8):e1003227. doi: 10.1371/journal.pmed.1003227 (PMC7428052; doi:10.1371/journal.pmed.1003227)
Supplement: S2 Appendix — (DOC) [file pmed.1003227.s012.doc]

**S2 Appendix**: Study questionnaire used during the malaria cross-sectional surveys conducted in Magude in Portuguese (original version), and translated into English.

**Estudo: Cross-sectional Study to Monitor *P.falciparum* transmission intensity in Southern Mozambique**

**Inquérito de estudo**

|  | Informação sócio-demográfica | | | | | | Nº de série | | | | | | |
| --- | --- | --- | --- | --- | --- | --- | --- | --- | --- | --- | --- | --- | --- |
|  | Número de estudo | | | | | | | XMAG-|__|__|__|__|__| | | | | | |
|  | Agregado | | | | | | | |__|__|__|__|-|__|__|__| | | | | | |
|  | Perm_ID do participante | | | | | | | |__|__|__|__|-|__|__|__|-|__|__| | | | | | |
|  | Data de nascimento | | | | | | | |__|__| - |__|__|__| - |__|__|__|__| | | | | | |
|  | Sexo | | | | | | | **□** Masculino **□** Feminino | | | | | |
|  | Visita foi feita? | | | | | | | **□** Sim **□** Não | | | | | |
|  | 1. Se não, especificar o motivo e passar para pergunta 42 | | | | | | | **□** Recusa **□** Ausente **□** Emigração  **□** Morte **□** Outro | | | | | |
|  | Data da visita | | | | | | | |__|__| - |__|__|__| - |__|__|__|__| | | | | | |
|  | Assinou o consentimento informado? | | | | | | | **□** Sim **□** Não | | | | | |
|  | Se o participante for uma mulher, está grávida? | | | | | | | **□** Sim **□** Não **□** Não sabe **□** NA | | | | | |
|  | 1. Se sim, paridade | | | | | | | **□** Primeira gravidez **□** Segunda ou mais | | | | | |
|  | Dados clínicos | | | | | | | | | | | | |
|  | Temperatura axilar | | | | | | | (99 se nao foi medida) |__|__|__|ºC | | | | | |
|  | História de febre nas últimas 24 horas? | | | | | | | **□** Sim **□** Não **□** Não sabe | | | | | |
|  | Teve febre durante os últimos 30 dias? | | | | | | | **□** Sim **□** Não **□** Não sabe | | | | | |
|  | 1. Se sim, foi ao centro/posto de saúde? | | | | | | | **□** Sim **□** Não **□** Não sabe | | | | | |
|  | Recebeu antimaláricos durante os últimos 30 dias? | | | | | | | **□** Sim **□** Não **□** Não sabe | | | | | |
|  | Se sim, que antimalárico (s) recebeu?  (marcar todos os possíveis)  (nas crianças ver o cartão amarelo) | | | | | | | **□** Eurartesim^2^  **□** CoArtem (8O5) **□** Fansidar (8O13 ou 8O14)  **□** Artesunato (8O6 ou 8O7) **□** Amodiaquina (8O1)  **□** Quinino (8O11 ou 8O12) **□** Não sabe  **□** Outro (especificar) _______________ | | | | | |
|  | Se **for Eurartesim** tomado durante a participação no estudo REACT, especificar a data do primeiro dia da toma^2^ | | | | | | | |__|__| - |__|__|__| (dd-mmm)  Não sabe | | | | | |
|  | Completou todas as vacinas do PAV correspondentes à idade da criança? (**só crianças <15 anos**) | | | | | | | **□** Sim **□** Não **□** Não tem cartão amarelo | | | | | |
|  | Medidas de controlo de malária | | | | | | | | | | | | |
|  | Participou na campanha de administração massiva de medicamentos de malária feita pelo CISM?^1^ | | | | | | | **□** Sim **□** Não | | | | | |
|  | 1. Se sim, qual foi a última actividade na que participou? ^1^   (Se possível pedir o último CI para apurar em que estudo participou). | | | | | | | **□** MDA (Dez 2016 / Janeiro 2017)  **□** REACT (Fevereiro-Maio 2017) | | | | | |
|  | 1. Se Sim, tomou o medicamento?^1^ | | | | | | | **□** Sim **□** Não | | | | | |
|  | 1. Se Sim, a quanto tempo tomou o medicamento?^1^ | | | | | | | |__|__| Meses | | | | | |
|  | A casa foi fumigada nos últimos 12 meses? | | | | | | | **□** Sim **□** Não **□** Não sabe | | | | | |
|  | 1. Se não, motivo. | **□** Equipa não veio **□** Não estavam em casa **□** Recusaram **□** Não sabe/outros | | | | | | | | | | | |
|  | Data da última pulverização (99-999-9999=não sabe) | | | | | | | |__|__| - |__|__|__| - |__|__|__|__| | | | | | |
|  | A pessoa dormiu em baixo de uma rede mosquiteira ontem? | | | | | | | **□** Sim **□** Não **□** Não sabe | | | | | |
|  | **Se sim, pedir para ver a rede que usa o participante.** | | | | | | |  | | | | | |
|  | 1. Se sim, é de impregnação de longa duração ou foi impregnada nos últimos 12 meses? | | | | | | | **□** Sim **□** Não **□** Não sabe | | | | | |
|  | 1. Quando adquiriram a rede? | | **□** Menos de 1 ano □ 1 a 2 anos **□** 2 a 3 anos **□** 3 anos ou mais  **□** Não sabe | | | | | | | | | | |
|  | 1. Onde adquiriram a rede? | | **□** Comprada □ Distribuída no centro de saúde ou pelo programa nacional  **□** Distribuída pelo CISM **□** Outro/não sabe | | | | | | | | | | |
|  | Quantas redes mosquiteiras penduradas tem no agregado? | | | | | | | | |__|__| | | | | |
|  | Alguma destas medidas foi usada na casa durante a última semana? (marcar todos os possíveis) | | | | | | | | **□** Espirais (dragão) **□** Repelentes  **□** Insecticida (baygon) **□** Nenhuma | | | | |
|  | **Se for uma mulher** grávida ou com uma gravidez durante os últimos 2 anos, recebeu TIP (tratamento intermitente presuntivo de malária) na consulta pré-natal? | | | | | | | | | | | **□** Sim **□** Não **□** Não sabe | |
|  | 1. Se sim, por quantas vezes? | | | | | | | | **□** Uma **□** Duas **□** Três **□** Não sabe | | | | |
|  | Dados sócio-económicos | | | | | | | |  | | | | |
|  | **Dados do participante ou da mãe/responsável se for uma criança (<16 anos)** | | | | | | | | | | | | |
|  | Nível de escolaridade formal | | | **□** Nenhum **□** EP1 **□** EP2 **□** Secundária **□** Médio **□** Superior | | | | | | | | | |
|  | Sabe ler e escrever? | | | **□** Sim **□** Não **□** Não sabe responder | | | | | | | | | |
|  | Ocupação (NA se não aplicável) | | | **□** Doméstico **□** Camponês **□** Camponês remunerado  **□** Estudante **□** Negociante **□** Serviços **□** Outro **□** NA | | | | | | | | | |
|  | Deixou de fazer a sua ocupação no último mês por doença? | | | **□** Sim **□** Não | | | | | | | | | |
|  | 1. Se sim, quantos dias deixou de trabalhar? | | | | | | | | | | | | |__|__|__| dias |
|  | Situação marital | | | | **□** Casado/a **□** União **□** Solteiro/a **□** Separado/a **□** Viúvo/a | | | | | | | | |
|  | 1. Seé casado/a ou vive em união: vive permanentemente com o parceiro? | | | | | | | | | | **□** Sim **□** Ocasionalmente **□** Não | | |
|  | **Assinatura inquiridor** | | | | | | | | | | | | |
|  | Código, assinatura e data | | |__|__|__| _____________________ | | | | | | | |__|__| - |__|__|__| - |__|__|__|__| | | | |
|  | Colheita de amostras | | | | | | | | | | | | |
|  | Teste rápido de malária (RDT) | | | | | **□** Positivo **□** Negativo **□** Inválido **□** Não feito | | | | | | | |
|  | Teste rápido de malária sensível (HS-RDT) ^1^ | | | | | **□** Positivo **□** Negativo **□** Inválido **□** Não feito | | | | | | | |
|  | Foram colhidas 2 lâminas? | | | | | **□** Sim **□** Não | | | | | | | |
|  | Foram colhidos 2 papéis de filtro? | | | | | **□** Sim **□** Não | | | | | | | |
|  | 1. Se sim, colar o NIDA e preencher folha de registo de laboratório | | | | |  | | | | | | | |
|  | Resultado do teste de gravidez (para mulheres de15-49 anos com teste rápido de malária positivo) | | | | | | | | **□** Positivo **□** Negativo **□** Recusa **□** NA | | | | |
|  | **Assinatura pessoal de laboratório** | | | | | | | | | | | | |
|  | Código, assinatura e data | | |__|__|__| _____________________ | | | | | | | |__|__| - |__|__|__| - |__|__|__|__| | | | |

**Controle de versões**

1. Perguntado em 2017
2. Perguntado em 2018

**Study: Cross-sectional Study to Monitor *P.falciparum* transmission intensity in Southern Mozambique**

**Survey questionnaire**

(English translation)

|  | Socio-demographic information | | | | | Serial number | | | | | | | | | | | | |
| --- | --- | --- | --- | --- | --- | --- | --- | --- | --- | --- | --- | --- | --- | --- | --- | --- | --- | --- |
|  | Study number | | | | | XMAG-|__|__|__|__|__| | | | | | | | | | | | | |
|  | Household number | | | | | |__|__|__|__|-|__|__|__| | | | | | | | | | | | | |
|  | Permanent ID number of the participant | | | | | |__|__|__|__|-|__|__|__|-|__|__| | | | | | | | | | | | | |
|  | Date of birth | | | | | |__|__| - |__|__|__| - |__|__|__|__| | | | | | | | | | | | | |
|  | Sex | | | | | **□** Male **□** Female | | | | | | | | | | | | |
|  | Was the visit done? | | | | | **□** Yes **□** No | | | | | | | | | | | | |
|  | 1. If no, specify the reason and go to the signatures section | | | | | **□** Refuses **□** Absent **□** Emigration  **□** Death **□** Other | | | | | | | | | | | | |
|  | Date of the visit | | | | | |__|__| - |__|__|__| - |__|__|__|__| | | | | | | | | | | | | |
|  | Did the participant sign an informed consent? | | | | | **□** Yes **□** No | | | | | | | | | | | | |
|  | If the participant is a woman, is she pregnant? | | | | | **□** Yes **□** No **□** Does not know **□** NA | | | | | | | | | | | | |
|  | 1. If yes, specify parity | | | | | **□** First pregnancy **□** Second or more pregnancies | | | | | | | | | | | | |
|  | Clinical data | | | | | | | | | | | | | | | | | |
|  | Axillary temperature | | | | | (99 if not measured) |__|__|__|ºC | | | | | | | | | | | | |
|  | History of fever in the previous 24 hours | | | | | **□** Yes **□** No **□** Does not know | | | | | | | | | | | | |
|  | History of fever in the previous 30 days | | | | | **□** Yes **□** No **□** Does not know | | | | | | | | | | | | |
|  | 1. If yes, did s/he go to the health center? | | | | | **□** Yes **□** No **□** Does not know | | | | | | | | | | | | |
|  | History of antimalarial intake in the previous 30 days | | | | | **□** Yes **□** No **□** Does not know | | | | | | | | | | | | |
|  | If yes, what antimalarials did s/he take?  (select all that apply)  (for children: ask to see the health card) | | | | | **□** Eurartesim (DHAp)^2^  **□** CoArtem (8O5) **□** Fansidar (8O13 ou 8O14)  **□** Artesunate (8O6 ou 8O7) **□** Amodiaquine (8O1)  **□** Quinine (8O11 ou 8O12) **□** Does not know  **□** Other (specify) _______________ | | | | | | | | | | | | |
|  | If s/he took Eurartesim during the reactive focal MDA activities (REACT), when?^2^ | | | | | |__|__| - |__|__|__| (dd-mmm)  Does not know | | | | | | | | | | | | |
|  | Has the child completed the vaccinations required for his/her age according to the EPI plan? (**for children <15 years-old**) | | | | | **□** Yes **□** No  **□** Does not have the child health card | | | | | | | | | | | | |
|  | Malaria prevention measures | | | | | | | | | | | | | | | | | |
|  | Did the participant participate in the mass drug administration campaign conducted by CISM this year?^1^ | | | | | **□** Yes **□** No | | | | | | | | | | | | |
|  | 1. If yes, when was the last time that the person participated? ^1^   (If possible, ask for the last informed consent copy signed). | | | | | **□** MDA (Dec 2016 / Jan 2017)  **□** REACT (February to May of 2017) | | | | | | | | | | | | |
|  | 1. If yes, did the participant take the drug?^1^ | | | | | **□** Yes **□** No | | | | | | | | | | | | |
|  | 1. If yes, how long ago was the last dose taken?^1^ | | | | | |__|__| Months | | | | | | | | | | | | |
|  | Was the house sprayed in the preceding 12 months? | | | | | **□** Yes **□** No **□** Does not know | | | | | | | | | | | | |
|  | 1. If no, why not? | **□** The team did not come **□** They were not home **□** Refused **□** Does not know / Other | | | | | | | | | | | | | | | | |
|  | Date of the last IRS event (99-999-9999=if s/he does not know) | | | | | | | |__|__| - |__|__|__| - |__|__|__|__| | | | | | | | | | | |
|  | Did the participant sleep under a bed net the night before? | | | | | | | **□** Yes **□** No **□** Does not know | | | | | | | | | | |
|  | **If yes, ask to see the net used** | | | | | | | |  | | | | | | | | | |
|  | 1. If yes, is the net a long-lasting insecticide treated net (LLIN) or has it been impregnated with an insecticide in the preceding 12 months? | | | | | | | | | | | | | **□** Yes **□** No **□** Does not know | | | | |
|  | 1. When was the bed net acquired? | | **□** Less than one year ago □ 1 to 2 years ago **□** 2 to 3 years ago **□** 3 or more years ago **□** Does not know | | | | | | | | | | | | | | | |
|  | 1. Where was the net acquired? | | **□** Purchased □ Distributed by the health center of national malaria control program **□** Distributed by CISM **□** Other / Does not know | | | | | | | | | | | | | | | |
|  | How many hanging nets are there in the household? | | | | | | | | | |__|__| | | | | | | | | |
|  | Was any of these measures used in the household in the last few weeks? (select all that apply) | | | | | | | | | | **□** Incense **□** Repellents  **□** Insecticides (Baygon) **□** None | | | | |  | | |
|  | **If the participant is pregnant or if she has had a pregnancy in the last 2 years**, did she receive IPTp during the ante-natal clinic visits? | | | | | | | | | | **□** Yes **□** No **□** Does not know | | | | | | |  |
|  | 1. If yes, how many times? | | | | | | | | | | **□** Once **□** Twice **□** Three times  **□** Does not know | | | | |  | | |
|  | Socio-economic data | | | | | | | | | |  | | | | | |  | |
|  | **Participants information, or information of the participant’s mother if the participant is <16 years old** | | | | | | | | | | | | | | | | | |
|  | Formal education level | | | **□** None **□** EP1 **□** EP2 **□** Secondary school **□** High-school **□** Higher-level education | | | | | | | | | | | | | | |
|  | Can the participant read and write? | | | **□** Yes **□** No **□** Does not know how to respond | | | | | | | | | | | | | | |
|  | Occupation (NA if not applicable) | | | **□** Stay-at-home parent **□** Farmer **□** Employed farmer  **□** Student **□** Salesperson **□** Public services **□** Other **□** NA | | | | | | | | | | | | | | |
|  | Did the person stop working at any time in the last month due to illness? | | | | | | | | | | | | | | **□** Yes **□** No | | | |
|  | 1. If yes, for how many days? | | | | | | | | | | | | | | |__|__|__| days | | | |
|  | Marital status | | | **□** Married **□** Civil union **□** Single **□** Separated **□** Widow | | | | | | | | | | | | | | |
|  | 1. If married or in civil union, does the participant live with his/her partner? | | | | | | | | | | | | **□** Yes **□** Occasionally **□** No | | | | | |
|  | **Data collector’s signature** | | | | | | | | | | | | | | | | | |
|  | Code, signature, date | | |__|__|__| _____________________ | | | | | | | | | |__|__| - |__|__|__| - |__|__|__|__| | | | | | | |
|  | Sample collection | | | | | | | | | | | | | | | | | |
|  | Malaria rapid diagnostic test (RDT) result | | | | **□** Positive **□** Negative **□** Invalid **□** Not done | | | | | | | | | | | | | |
|  | High-sensitive RDT result (HS-RDT) ^1^ | | | | **□** Positive **□** Negative **□** Invalid **□** Not done | | | | | | | | | | | | | |
|  | Were 2 blood smears collected? | | | | | | | | | | | **□** Yes **□** No | | | | | | |
|  | Were 2 filter papers collected? | | | | | | | | | | | **□** Yes **□** No | | | | | | |
|  | 1. If yes, stick the sample ID sticker (or type the number) | | | | | | | | | | |  | | | | | | |
|  | Pregnancy RDT result (for women aged 15-49 with a positive malaria RDT result) | | | | | | **□** Positive **□** Negative **□** Refuses **□** NA | | | | | | | | | | | |
|  | **Signature of the laboratory technician** | | | | | | | | | | | | | | | | | |
|  | Code, signature and date | | |__|__|__| _____________________ | | | | | | | | | |__|__| - |__|__|__| - |__|__|__|__| | | | | | | |

**Version control**

1. Question made in 2017
2. Question made in 2018
