# Supplementary material for: A multiphase program for malaria elimination in southern Mozambique (the Magude project): A before-after study
Source: PLoS Med. 2020 Aug 14;17(8):e1003227. doi: 10.1371/journal.pmed.1003227 (PMC7428052; doi:10.1371/journal.pmed.1003227)
Supplement: S3 Appendix — ITS, interrupted time series. (DOCX) [file pmed.1003227.s013.docx]

**S3 Appendix:** Interrupted time series model structure and description

Y_t_ = β_0_ + β_1_T_t_ + β_2_Int_1_ + β_3_Int_1_*T_t_ + β_4_Int_2_ + β_5_Int_2_*T_t_ + Σβ_j_Covariates + ε_t_

**Data structure**

| *T* | *Int_1_* | *Int_1_*T* | *Int_2_* | *Int_2_*T* | Covariate _j_ |
| --- | --- | --- | --- | --- | --- |
| 1 | 0 | 0 | 0 | 0 | n_t_ |
| 2 | 0 | 0 | 0 | 0 | n_t_ |
| 3 | 0 | 0 | 0 | 0 | n_t_ |
| 4 | 1 | 0 | 0 | 0 | n_t_ |
| 5 | 1 | 1 | 0 | 0 | n_t_ |
| 6 | 1 | 2 | 0 | 0 | n_t_ |
| 7 | 1 | 3 | 1 | 0 | n_t_ |
| 8 | 1 | 4 | 1 | 1 | n_t_ |
| 9 | 1 | 5 | 1 | 2 | n_t_ |
| 10 | 1 | 6 | 1 | 3 | n_t_ |

**Model variables**

- *Yt* is the dependent variable and represents the number of weekly (*t*) cases of malaria detected by microscopy or RDT at the health facilities or by the community health workers
- *Tt* is the number of weeks since the beginning of the study
- *Int_1_* is a dummy variable representing phase I intervention (pre-intervention period as 0, post-intervention period as 1)
- *Int_1_*T_t_* is an interaction term (phase I)
- *Int_2_* is a dummy variable representing phase II interventions
- *Int_2_*T_t_* is an interaction term (phase II)
- Covariates used in the estimation of the coefficients were temperature (1 month lag), rainfall (1 month lag), EVI (1 month and 2 months lag), non-malaria cases, LLINs per capita (1 month lag). The latter included a linear integrity decay function assuming 100% integrity within the first six months after the 2014 distribution, a 20% reduction in the number of fully functioning nets after two years (based on a net integrity assessment conducted in Magude), and an extension of this linear decay until the 2017 net distribution.

**Model coefficients** [1].**:**

- *β_0_* represents the starting level of the dependent variable
- *β_1_* is the slope until the introduction of phase I interventions
- *β_2_* is the change in the level that occurs in the period immediately following the introduction of phase I interventions (immediate effect of phase I interventions)
- *β_3_* is the difference between preintervention and phase I slopes (effect over time of phase I interventions compared with preintervention)
- *β_4_* is the change in the level that occurs in the period immediately following the introduction of the phase II interventions (immediate effect of phase II interventions)
- *β_5_* is difference between phase I and phase II slopes (effect over time of phase II interventions compared with phase I)
- *ε_t_* the error term.

**References**

1. Linden A. Conducting interrupted time-series analysis for single- and multiple-group comparisons. The Stata Journal. 2015;15:480–500.
